# Fourier transform infrared spectroscopy enables rapid strain typing in *M. pachydermatis*

**DOI:** 10.1128/jcm.01422-25

**Published:** 2026-07-20

**Authors:** Simon Kurmann, Marco A. Coelho, Márcia David-Palma, Leyna Díaz, Gemma Castellá, F. Javier Cabañes, Joseph Heitman, Salomé LeibundGut-Landmann, Francis Muchaamba

**Affiliations:** 1 Section of Immunology, Vetsuisse Faculty, University of Zurich 30843, Zurich, Switzerland; 2 Department of Molecular Genetics and Microbiology, Duke University School of Medicine 242247, Durham, North Carolina, USA; 3 Veterinary Mycology Group, Department of Animal Health and Anatomy, Veterinary School, Universitat Autònoma de Barcelona 571800, Bellaterra, Spain; 4 Institute of Experimental Immunology, University of Zurich 31010, Zurich, Switzerland; 5 Medical Research Council Centre for Medical Mycology at the University of Exeter, Department of Biosciences, Faculty of Health and Life Sciences 98452, Exeter, United Kingdom; 6 Institute for Food Safety and Hygiene, Vetsuisse Faculty, University of Zurich 600627, Zurich, Switzerland; University of California Davis, Davis, California, USA

**Keywords:** *Malassezia pachydermatis*, Fourier transform infrared (FTIR) spectroscopy, molecular typing, strain discrimination

## Abstract

*Malassezia pachydermatis* is a zoophilic yeast found on the skin and in the outer ear canal of many mammals. It normally maintains a commensal lifestyle, but can cause dermatitis and otitis in predisposed hosts, particularly in atopic dogs. *M. pachydermatis* is genetically diverse, with strains clustering into at least three phylogroups based on molecular typing, a pattern we now confirm through whole-genome sequencing (WGS). Accurate species and strain-level identification is essential for understanding its epidemiology, pathogenic potential, and response to treatment. In this study, we established Fourier transform infrared (FTIR) spectroscopy as a rapid, cost-effective method for distinguishing *M. pachydermatis* from other *Malassezia* species, including *M. globosa*, *M. furfur*, *M. restricta*, and *M. sympodialis*. Within *M. pachydermatis*, FTIR spectroscopy resolved even closely related strains with high accuracy, producing clusters congruent with multilocus sequence typing (MLST)- and WGS-based phylogeny. The incorporation of an artificial neural network classifier further enhanced the discriminatory power, enabling robust and automated strain assignment. These findings demonstrate the potential of FTIR spectroscopy as a practical tool for large-scale epidemiological surveillance of *M. pachydermatis* and for clinical and veterinary applications where strain-level identification could inform treatment and management of *Malassezia*-associated diseases.

**IMPORTANCE:**

*Malassezia pachydermatis* is a yeast that commonly inhabits the skin and ear canals of mammals but can cause dermatitis and otitis in predisposed hosts, especially dogs with allergies or immunosuppression. This species displays substantial genetic diversity, with strains falling into distinct phylogroups that may differ in their biology and clinical significance. Determining these differences has typically required advanced molecular or genomic methods, which can be costly and time-consuming. In this study, we demonstrate that Fourier transform infrared spectroscopy can rapidly and accurately distinguish *M. pachydermatis* from other *Malassezia* species and resolve genetic groups within the species in a way that reflects whole-genome relationships. This capability offers a practical tool for investigating the epidemiology and inter-/intraspecies diversity of *M. pachydermatis* and for guiding targeted management of *Malassezia*-associated diseases in both veterinary and, potentially, human medicine.

## INTRODUCTION

*Malassezia* yeasts, a genus of lipophilic basidiomycetes, are found on the skin of warm-blooded animals and humans (1, 2). While usually harmless, they can act as opportunistic pathogens and, as such, are implicated in various skin disorders such as dandruff, seborrheic dermatitis, and atopic dermatitis in humans (3), and similar conditions in animals (2). On the other hand, *Malassezia* also undergoes mutual interactions with the host by enhancing the barrier integrity of the skin (4) and by antagonizing skin bacteria to increase colonization resistance (5, 6).

To date, 18 culturable species of *Malassezia* have been identified (7, 8), with *M. pachydermatis* representing the predominant species in many domestic animals (1, 2, 9, 10) and also in wild animals (11, 12). In healthy dogs, regardless of the breed, *M. pachydermatis* is found in the ear canal and the perioral and interdigital regions (2). Dogs with otitis externa and/or dermatitis have a higher abundance of *M. pachydermatis*. In addition, isolates from diseased dogs were found to exhibit elevated phospholipase A2 activity (13–16), which aligns with the idea that *Malassezia* contributes to pathogenesis by hydrolyzing sebum triglycerides into free fatty acids, including some that trigger inflammation and skin barrier disruption (17).

Besides its primarily zoophilic nature, *M. pachydermatis* has also zoonotic potential. Rare cases of life-threatening fungemia in humans have been traced back to pet dogs, especially in healthcare settings where hospital workers may act as intermediaries (18). *Malassezia* fungemia occurs primarily in critically ill, immunocompromised patients, especially premature infants receiving lipid-rich parenteral nutrition, and manifests with fever, chills, and lethargy mimicking bacterial sepsis (19). Although *Malassezia* is not typically considered a major public health risk, its presence in hospital environments and the rising antifungal resistance (20) has raised concerns (19).

*M. pachydermatis* displays a high genetic diversity (1, 10, 12, 21, 22). While the clinical significance of this diversity is unclear, it likely affects the fungus’ interaction with the host, reminiscent of what has been described for other opportunistic fungal pathogens such as *Candida albicans* (23). Genetic diversity within *M. pachydermatis* has been explored through multilocus sequence typing (MLST) (1, 10, 24, 25). More recently, *M. pachydermatis* has been sequenced (26). However, only very few complete genomes are currently publicly available (18) for analyzing the intraspecies diversity at the genomic level. This scarcity of complete genomic data hinders efforts to fully understand the population structure, evolutionary dynamics, and potential genotype–phenotype associations of *M. pachydermatis*, highlighting the need for additional high-quality genome sequences.

Fourier transform infrared (FTIR) spectroscopy offers a compelling alternative for identifying microbial strain variations, with high discriminatory power, as shown for several bacterial (27, 28) and fungal pathogens, including *Candida*, *Cryptococcus,* and dermatophytes (29–33) as well as phytopathogens (34–36). However, it has not yet been explored for *Malassezia* species. The method is based on quantifying the absorption of infrared light by cellular biomolecules such as nucleic acids, proteins, lipids, and carbohydrates. The interaction of these molecules with infrared light generates a highly specific absorption signature, often referred to as a biochemical fingerprint, characteristic of the organism or strain, and can thus be used for cluster analysis and organism typing (27). Due to its cost- and time-effectiveness, FTIR spectroscopy holds promise for improving the speed and accuracy of strain typing in research and diagnostics.

In this study, we established FTIR spectroscopy as a reliable method for *M. pachydermatis* typing. We show that this method effectively distinguishes *M. pachydermatis* from other *Malassezia* species, discriminates between *M. pachydermatis* strains, and clusters them in a manner comparable to hierarchical clustering based on MLST and whole-genome sequencing (WGS) data. Furthermore, we established a standardized classification system based on the strains’ spectral signatures.

## MATERIALS AND METHODS

### Fungal strains

*M. pachydermatis* (*n* = 30) haploid strains, collected between 1995 and 2018, were selected to represent the species’ genetic diversity (Table 1). Strains from other *Malassezia* species (*M. furfur* [*n* = 12], *M. globosa* [*n* = 2], *M. sympodialis* [*n* = 6], and *M. restricta* [*n* = 1]), and from *C. albicans* (*n* = 2), were also included (Table 2).

**TABLE 1:** *M. pachydermatis* strains used in this study

Strain^*a*^	Country	Isolation site	Isolation date	Phylogroup^*b*^	Reference	Classifier^*c*^
CBS1879^*d*^	Sweden	Ear	n/a	I	(10)	Validation
MA94	Spain	Groin	n/a	I	(10)	Training
MA1595	Spain	Mammary gland	2018	I	(37)	Validation
MA872	Spain	Ear	2006	I	(37)	Validation
MA587	Spain	Ear	2001	I	(37)	Validation
MA860	Spain	Ear	2005	I	(37)	Training
KCTC27587	Korea	Ear	n/a	I	(38)	Training
MA1398	Spain	Skin	2010	I	(37)	Training
MA636	Spain	Skin	2003	I	This study	Validation
MA1239	Spain	Ear	2009	I	(37)	Validation
MA1401	Spain	Ear	2010	I	(37)	Validation
MA1441	Spain	Ear	2011	I	(37)	Training
MA506	Spain	Ear	2001	II	(37)	Training
MA724	Spain	Skin	2004	II	This study	Validation
MA718	Spain	Ear	2004	II	This study	Training
MA1486	Spain	Skin	2015	II	This study	Training
MA440	Spain	Ear	1999	II	This study	Validation
MA688	Spain	Ear	2004	II	(37)	Validation
MA8	Spain	Ear	1995	II	(37)	Validation
MA1195	Spain	Ear	2008	II	(37)	Validation
MA165	Spain	Ear	1997	II	(37)	Validation
MA1231	Spain	Skin	2009	II	(37)	Validation
MA1285	Spain	Skin	2009	II	(37)	Validation
MA259	Spain	Ear	1998	II	(37)	Training
MA9	Spain	Ear	1995	III	This study	Training
MA158	Spain	Ear	1997	III	This study	Training
MA280	Spain	Ear	1998	III	(10)	Training
MA1289	Spain	Interdigital fold	2009	III	(37)	Validation
MA361	Spain	Ear	1999	III	(37)	Training
MA1174	Spain	Ear	2008	III	(37)	Validation

^*a*^ CBS, Centraal Bureau voor Schimmelcultur; MA, culture collection of the Veterinary Mycology group, Universitat Autònoma de Barcelona, Bellaterra, Catalonia, Spain; KCTC, Korean Collection of Type Cultures.

^*b*^ Phylogroup assignment based on WGS.

^*c*^ Strains labeled as “Training” were used to train the FTIR artificial neural networks (ANN) classifier, while those labeled as “Validation” were used for its validation.

^*d*^ Internal strain code: M2.

**TABLE 2:** Other yeast strains used in this study

Species	Strain ID	Internal strain code	Host species	Reference
*M. furfur* ^*a*^	CBS1878	M1	human	(25)
*M. furfur* ^*a*^	CBS7019	M88	human	(39)
*M. furfur* ^*b*^	CD866	M75	human	(40)
*M. furfur* ^*b*^	CBS9595	M77	human	(40)
*M. furfur* ^*c*^	CBS14141	M7	human	(40)
*M. furfur* ^*c*^	PM315	M78	human	(40)
*M. furfur*	GS1B	M38	human	(41)
*M. furfur*	GS2A	M39	human	(41)
*M. furfur*	GS2B	M35	human	(41)
*M. furfur*	B22	M34	human	(41)
*M. furfur*	B412	M37	human	(41)
*M. furfur*	CBS9596	M36	human	(42)
*M. globosa*	CBS7966	M3	human	(25)
*M. globosa*	GB_AD72_270223	M52	human	this study
*M. sympodialis*	ATCC 42132	M67	human	(43)
*M. sympodialis*	10102 (KS012)	M26	human	(44)
*M. sympodialis*	10103 (KS013)	M27	human	(44)
*M. sympodialis*	10104 (KS014)	M55	human	(44)
*M. sympodialis*	10105 (KS015)	M57	human	(44)
*M. sympodialis*	10107 (KS017)	M29	human	(44)
*M. restricta*	RS HS_19	M49	human	this study
*C. albicans*	SC5314		human	(45)
*C. albicans*	101		human	(23)

^*a*^ Hybrid.

^*b*^ Haploid, P1 lineage.

^*c*^ Haploid, P2 lineage.

### Culture conditions

All *Malassezia* strains were preserved in glycerol at −80°C and revived by transferring an aliquot on modified Dixon (mDixon) agar (36 g malt extract (Sigma), 20 g desiccated oxbile (Sigma), 6 g bacto peptone (Becton Dickinson), 10 mL Tween 40 (Sigma), 2 mL pure glycerol (Sigma), 2 mL oleic acid (Sigma, stored at −20°C), and 15 g agar [Sigma] per liter, pH adjusted to 6.0 using HCl) and incubating the plates for 2–5 days at 30°C. *C. albicans* strains were grown on YPD agar (20 g D-glucose [Cayman Chemicals], 10 g Bacto yeast extract [Becton Dickinson], 20 g Bacto peptone, 20 g agar per liter) for 24 hours at 30°C. For FTIR analysis, standardized cultivation conditions were applied. Individual fungal colonies were sub-cultured on fresh mDixon agar for exactly 48 hours at 30°C. *M. restricta* was incubated for 7 days due to its slow growth rate.

### FTIR spectroscopy

FTIR spectroscopy analysis was performed using the IR Biotyper (Bruker Daltonics), following the manufacturer’s instructions with minor modifications. For sample preparation, two 1 µL inoculation loops of biomass collected from colonies grown on mDixon agar were transferred into 70 µL of deionized water in a 1.5 mL tube containing metal rods (Bruker Daltonics). The suspension was homogenized by shaking on an MSC 100 Thermo Shaker (Labgene Scientific) at 1,400 rpm and room temperature for 10 minutes. Subsequently, 70 µL of 70% (vol/vol) ethanol was added, followed by an additional 5 minutes of shaking. For initial colony resuspension, the reverse was also tried, with ethanol added first, followed by water. In this case, two 1 µL inoculation loops of biomass were transferred into 70 µL of 70% (vol/vol) ethanol in a 1.5 mL tube containing metal rods, homogenized by shaking at 1,400 rpm for 10 minutes. Then, 70 µL of deionized water was added, and the suspension was shaken for an additional 5 minutes. However, as this ethanol-first method resulted in poor and inconsistent homogenization of *Malassezia* colonies, the water-first resuspension protocol was adopted for all downstream analyses.

Next, 15 µL of the fungal suspension was spotted in triplicate on a silicon-based 96-sample plate (Bruker Daltonics) and allowed to dry at 37°C. An amount of 10 μL of the reconstituted Infrared Test Standards (IRTS 1 and IRTS 2) was also spotted in duplicate onto the IR Biotyper target and let dry as described for the samples. Spectra acquisition was performed using the IR Biotyper system in transmission mode over a spectral range of 4,000–500 cm⁻¹ using OPUS software v8.2 (Bruker Optics) with default analysis settings. Three independent biological repeats (independent colonies from independent plates), with at least three technical replicates each, were conducted for each strain, resulting in at least nine spectra per strain. Reproducibility was assessed using independent subcultures prepared on three separate days for representative strains.

Data analysis was performed using IR Biotyper Client Software v4.0 (Bruker Daltonics). Only spectra that passed the software-intrinsic quality control were further analyzed. Spectra were pre-processed by smoothing using the Savitzky–Golay algorithm prior to second-derivative transformation, followed by spectral splicing and vector normalization (28). Spectral windows corresponding to proteins, lipids, polysaccharidores, or their combinations were evaluated to identify the most discriminatory region. Subsequently, spectral splicing methods covering the range characteristic for polysaccharides (1,300–800 cm⁻¹) were applied, unless specified differently. Clustering of spectra was performed using hierarchical cluster analysis (HCA), principal component analysis (PCA), and linear discriminant analysis (LDA) for both supervised and unsupervised classification. Euclidean distance and average linkage clustering methods were applied to define clusters, using the determined Cut-Off Values (see below). Results were visualized through dendrograms, distance matrices, and 2D and 3-D scatter plots.

Starting from the cut-off values automatically generated by the software, an optimization procedure was conducted to determine the optimal values for *M. pachydermatis*, tailored to our laboratory-specific conditions. This optimization was informed by the genetic relatedness of the strains, as determined by MLST and WGS (see below).

### Genomic DNA extraction, genome sequencing and assembly

Genomic DNA was extracted from selected *M. pachydermatis* strains using a phenol:chloroform-based protocol as previously described (46). DNA concentrations were quantified with the Qubit dsDNA Assay Kit (Invitrogen) using a Qubit fluorometer. Whole-genome sequencing was performed at the Duke University Sequencing and Genomic Technologies (DUSGT) Core Facility. Libraries were prepared with the Kapa HyperPlus kit and sequenced on the Illumina NovaSeq platform to generate 2 × 150 bp paired-end reads. Draft genome assemblies were produced with SPAdes v3.15.3 (47), using default parameters and retaining contigs longer than 500 bp.

### Phylogenetic and average nucleotide identity analyses

Phylogenetic relationships were inferred from both genome-wide SNP data and MLST markers. For the whole-genome analysis, high-confidence single-nucleotide variants (SNVs) were called with Snippy v4.6.0, using the *M. pachydermatis* KCTC27587 genome as reference and the following parameters: *--cpus 10 --unmapped --mincov 10 --minfrac 0.9*. The resulting core genome alignment (30 sequences, 373,384 sites) served as input for maximum likelihood (ML) tree reconstruction with IQ-TREE v2.3.6, applying the following parameters: *-m MFP -msub nuclear --bnni -B 1000 -alrt 1000*, which enables model selection, nuclear-specific substitution models, and branch support via 1,000 ultrafast bootstrap (UFboot) replicates and the Shimodaira–Hasegawa approximate likelihood ratio test (SH-aLRT).

MLST-based phylogenies were reconstructed from both concatenated and single-locus alignments. Sequences of four loci previously shown to differentiate *M. pachydermatis* strains—large subunit (LSU; D1/D2 domain) and internal transcribed spacer (ITS) ribosomal RNA (rRNA) regions, *CHS2* (chitin synthase 2), and *TUB2* (β-tubulin)—were retrieved from each genome assembly via BLASTN searches using one deposited reference sequence per marker as query (10). For each locus, the top match per strain was extracted with *samtools faidx*, and reverse-strand hits were reoriented. The retrieved sequences were aligned with MAFFT v7.310 (*--adjustdirection*, FFT-NS-2 mode) and trimmed to the defined amplicon boundaries established in the original MLST scheme (10). In the concatenated analysis, the four trimmed alignments (30 sequences, 4 partitions, 5,434 sites) were merged into a partitioned supermatrix and analyzed with IQ-TREE v2.3.6 using the same ML parameters described above, with the *-p* option to define partitions. In parallel, single-gene ML trees were constructed to assess the phylogenetic resolution of each marker and to assign allele types. Each gene alignment was supplemented with published reference sequences representing known MLST alleles (10, 12, 37) (Table S1), and individual trees were inferred with IQ-TREE under the best-fit substitution model selected per gene, with 10,000 UFboot and SH-aLRT replicates to improve branch support resolution for the shorter alignments.

Pairwise average nucleotide identity (ANI) values were computed with OrthoANIu (48) (Tables S2 and S3). Custom Python scripts transformed ANI values into distance scores (100 − ANI), applied average linkage clustering, and generated a Newick-format dendrogram. Group-wise ANI statistics (minimum, maximum, mean, and number of comparisons) were derived from a strain-to-clade mapping file. All phylogenetic trees and dendrograms were visualized with iTOL v7.

### Comparison of FTIR and WGS-based clusters

Three different metrics were employed to compare clustering concordance between FTIR- and WGS-based approaches, calculated using an online tool (http://www.comparingpartitions.info/) with 95% confidence intervals (CIs) (49). The Simpson’s Index of Diversity (SID) was calculated separately for FTIR- and WGS-based clusterings to evaluate the diversity of clusters identified by each method. The Adjusted Rand Index (ARI) measured the degree of agreement between clustering methods while correcting for chance, with values closer to 1 indicating stronger concordance (49–51). Finally, the Adjusted Wallace Coefficient (AWC) quantified directional congruence between clustering methods. Specifically, the AWC quantifies the probability that two strains clustered together by one method (e.g., WGS) are also clustered together by the other (e.g., FTIR spectroscopy), and vice versa, with a value of 1 indicating complete agreement between two methods. Optimal phylogroup-specific FTIR cut-off values were defined by maximizing the ARI between FTIR- and ANI-based clustering.

### Development of an automated classifier for *M. pachydermatis*

To enable the automated classification of *M. pachydermatis* strains based on FTIR spectra, we developed a classifier using artificial neural networks (ANN) integrated within the IR Biotyper software. This classifier was designed to rapidly identify unknown samples based on spectral data, using marker models derived from a set of training spectra to categorize strains into three distinct *M. pachydermatis* phylogroups (I, II, and III). For classifier development, strains were divided into training and validation sets (Table 1). The training set, comprising five phylogroup I strains and four strains each from phylogroups II and III, was used to develop the classifier (*n* = 9 spectra per strain). The remaining 17 strains (*n* = 9 spectra per strain) formed the validation set for evaluating classifier performance. To ensure comprehensive representation of spectral variability, including outliers, training strains were selected based on deviation plots of the individual vector-normalized average spectrum of each strain within its respective phylogroup (Fig. S1A through C). Data from three biological replicates were used per strain to enhance the robustness and reproducibility of the classifier. Principal components (PCs) for training were selected using an overall vector-normalized spectral deviation plot of all three phylogroups (Fig. S1D), allowing exclusion of components that primarily contributed noise or non-informative variance. Training cycle parameters were optimized to avoid overfitting, aiming to produce a classifier applicable to real-world data. Classifier performance was evaluated using stratified fourfold cross-validation, with a confusion matrix generated to assess classification accuracy.

The classification results were reported using a scoring system with color coding to indicate confidence levels. A “green” score denoted a high-confidence match, where the sample spectrum fell within the spectral space of the training set (outlier value <1.0). A “yellow” score indicated moderate confidence, corresponding to spectra on the periphery of the spectral space of the training set (outlier value between 1.0 and 2.0). “Red” was assigned when the sample spectrum was outside the training set spectral space (outlier value >2.0), categorizing it as “no classification possible.” Misclassifications were defined as incorrect classifications assigned green or yellow scores. Classifier performance was evaluated based on accuracy (the proportion of correctly classified spectra), error rate (misclassified spectra/total spectra), sensitivity (the proportion of target strains correctly identified), specificity (the proportion of non-target strains correctly excluded), and positive and negative predictive values (Table S4). Additionally, classifier versatility was assessed using spectra from non-*M*. *pachydermatis* strains to evaluate its capacity to exclude unrelated species and its potential for broader application.

## RESULTS

### Phylogeny of *M. pachydermatis* strains based on MLST and WGS

**Fig 1:**
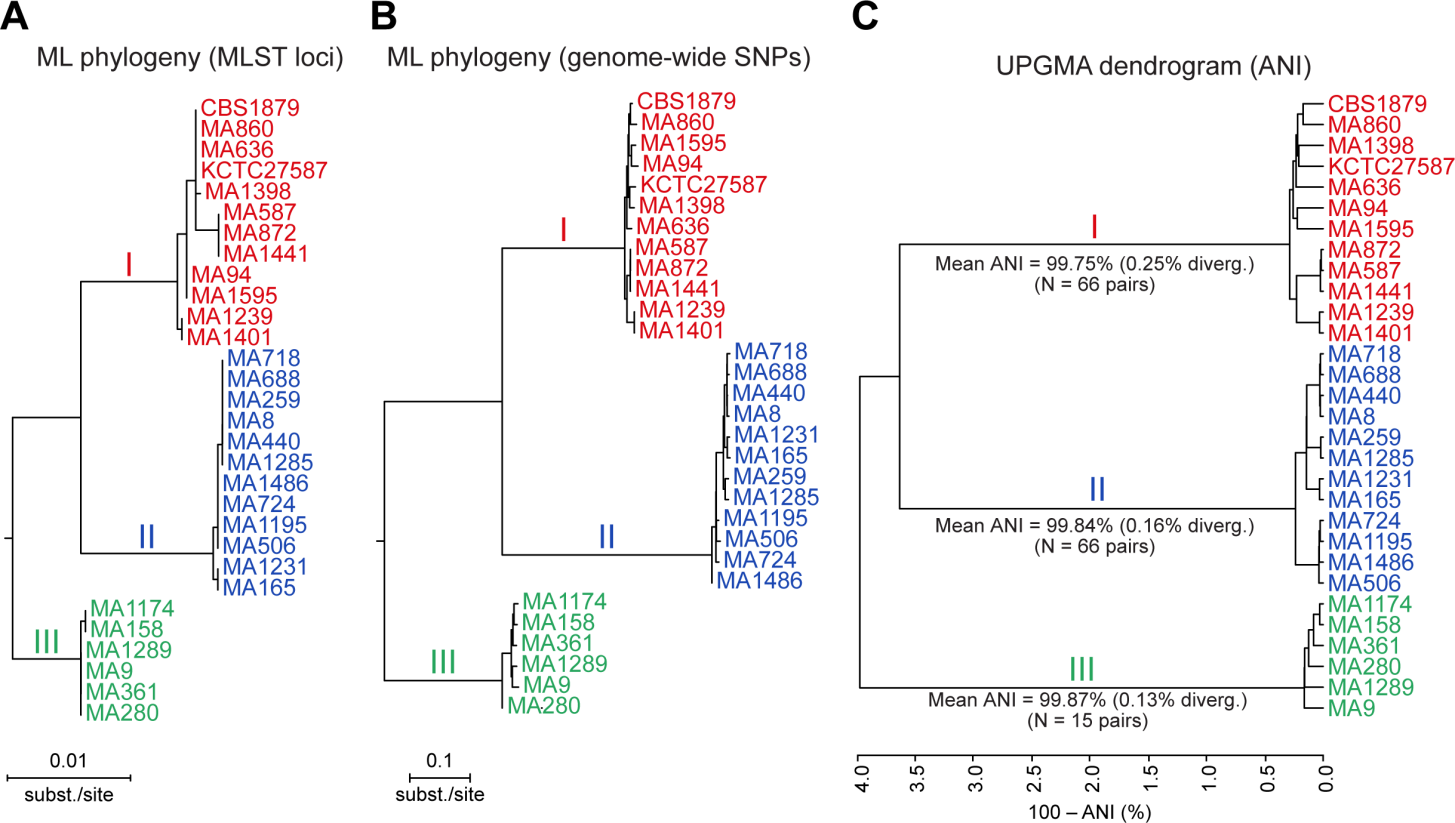
Phylogenetic relationships and sequence divergence among *M. pachydermatis* strains. (**A**) ML phylogeny based on a concatenated MLST alignment comprising 5,434 nucleotide sites across four loci: ITS1, LSU, *CHS2*, and *TUB2*. IQ-TREE was run with partitioned models. Branch support was assessed with the ultrafast bootstrap and SH-aLRT; 100% support was confirmed only for the three major phylogroups; internal nodes received low support and are not annotated for simplicity. (**B**) ML phylogeny inferred from 373,384 genome-wide single-nucleotide variants, based on the core genome alignment. Tree reconstruction was performed under the best-fit substitution model (TVM + F + ASC + R2; transversion model with empirical base frequencies, ascertainment-bias correction, and a FreeRate model with two rate categories) according to the Bayesian information criterion (BIC). All branches received 100% support from both ultrafast bootstrap and SH-aLRT tests. Both phylogenetic trees (**A and B**) were midpoint rooted. (**C**) Unweighted pair group method with arithmetic mean (UPGMA) dendrogram based on pairwise sequence divergence, calculated as 100 − ANI, with values computed by OrthoANIu. Mean ANI, corresponding average divergence, and the number of pairwise comparisons are indicated for each phylogroup. Strains are colored by phylogroup: group I (red, *n* = 12), group II (blue, *n* = 12), and group III (green, *n* = 6).

To investigate the genomic diversity of the 30 *M. pachydermatis* strains included in this study, we first performed MLST analysis with four loci—*CHS2*, LSU (D1/D2 domain of the large subunit rRNA), ITS, and *TUB2*—previously shown to differentiate *M. pachydermatis* (10), using sequences extracted from newly generated draft genome assemblies. The concatenated MLST phylogeny resolved the strains into three well-supported phylogroups, here designated I, II, and III (Fig. 1A; Fig. S2). Among the single-gene trees, the LSU provided the clearest separation of these groups, while ITS and *TUB2* showed higher levels of intra-group polymorphism. In addition to confirming previously defined allele types, our analysis identified two novel genotypes at the ITS (type XIX in strains MA1486, MA1195, MA724, MA506; and type XX in strains MA158 and MA1174) (Fig. S2), thereby expanding the documented allelic diversity of this species.

We next reconstructed phylogenetic relationships using WGS data to achieve higher resolution. A maximum-likelihood tree based on 373,384 high-confidence SNVs from the core genome closely agreed with the MLST results, confirming the assignment of all strains to the three phylogroups initially delineated by MLST, with all major nodes receiving 100% support in both ultrafast bootstrap and SH-aLRT tests (Fig. 1B).

Pairwise ANI values further highlighted differences in intra- and inter-clade diversity (Fig. 1C; Table S2). Phylogroup I (*n* = 12) exhibited the greatest heterogeneity, with a mean ANI of 99.75% corresponding to 0.25% average genomic divergence. Phylogroup II (*n* = 12) showed intermediate variability (mean ANI 99.84%; divergence 0.16%), whereas phylogroup III (*n* = 6) was the most homogeneous group (mean ANI 99.87%; divergence 0.13%). Comparative divergence between groups revealed that phylogroup I was genetically closer to phylogroup II (3.63%) than to phylogroup III (3.68%), while phylogroups II and III were the most divergent pair (4.26%) (Table S3).

Together, these results demonstrate that *M. pachydermatis* is structured into three well-supported genomic lineages, identifiable by both MLST and WGS. While MLST provides a useful framework for initial strain classification, WGS offers greater resolution, enabling robust phylogenetic inference and quantitative assessments of genomic divergence. These findings establish a phylogenetic framework for *M. pachydermatis*, providing the basis for future studies to explore how lineage-specific genomic variation may influence host interactions, pathogenic potential, or ecological adaptation.

### Establishing a working protocol for analyzing *Malassezia* by FTIR

While WGS provides high-resolution insights, its routine use remains limited; therefore, we explored FTIR spectroscopy as a high-throughput and cost-effective alternative for strain typing. For establishing a standardized protocol for FTIR analysis of *Malassezia* spp. using the IR Biotyper, we first developed a reliable sample preparation protocol that ensured that all strains consistently passed the initial quality control checks of the IR Biotyper software. To overcome the limited ability to resuspend *Malassezia* colony material in ethanol (Fig. S3), the fungal colony material was first resuspended in water, followed by the addition of an equal volume of 70% ethanol. This procedure contrasted with the manufacturer’s ethanol-first method, which, even after extended vortexing (>15 minutes), failed to yield homogeneous suspensions of colony material and resulted in poor-quality spectra that frequently failed the quality control check. Our modified “water-first, then ethanol” approach yielded FTIR spectra of high signal quality that consistently passed the IR Biotyper software’s quality control checks. The resulting spectra were highly reproducible across both technical and biological replicates of the strains. This optimized protocol was thus adopted as the default method for the entire study.

**Fig 2:**
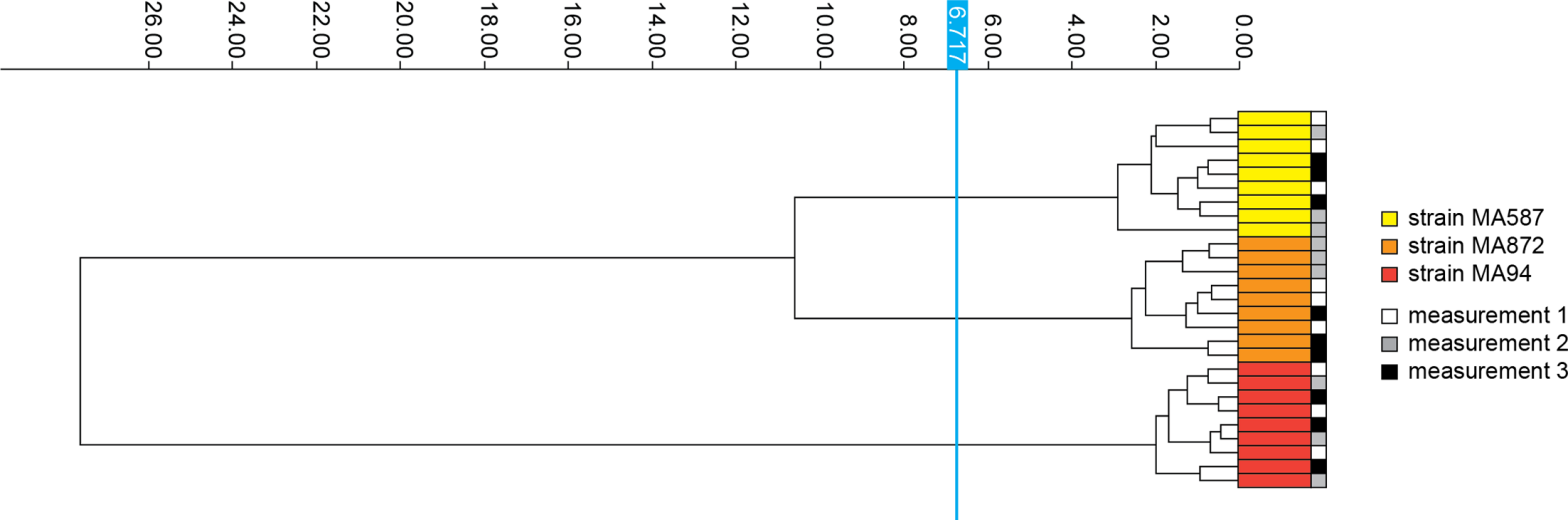
FTIR is highly reproducible across technical and biological replicates. Dendrogram generated from clustering FTIR spectra of *M. pachydermatis* strains (MA872, MA587, and MA94) to assess reproducibility across technical and biological replicates. Spectra are color-coded by strain and acquisition date, with each row representing a technical replicate derived from three independent experiments conducted on three separate days. The vertical blue line denotes the automatically calculated cut-off value (6.717) for clustering. Dimensionality reduction: LDA (2 LDs of 10 PCs; 95.7% variance).

To evaluate the reproducibility of the method, FTIR spectra of three *M. pachydermatis* strains were acquired on three different days with three technical replicates each. Both the technical and biological replicates of these strains clustered tightly for each strain (Fig. 2), highlighting the reproducibility and reliability of the protocol.

Comparison of the spectral ranges corresponding to polysaccharides (1,300–800 cm^−^¹), proteins (1,800–1,500 cm^−^¹), lipids (3,000–2,800 cm^−^¹, and 1,500–1,400 cm^−^¹), and polysaccharide–protein combinations (1,800–900 cm^−^¹) revealed that the splicing method for the polysaccharide region exhibited the highest discriminatory power (Fig. S4). Therefore, all subsequent analyses focused on the polysaccharide spectral region.

### FTIR spectroscopy discriminates between different *Malassezia* spp.

**Fig 3:**
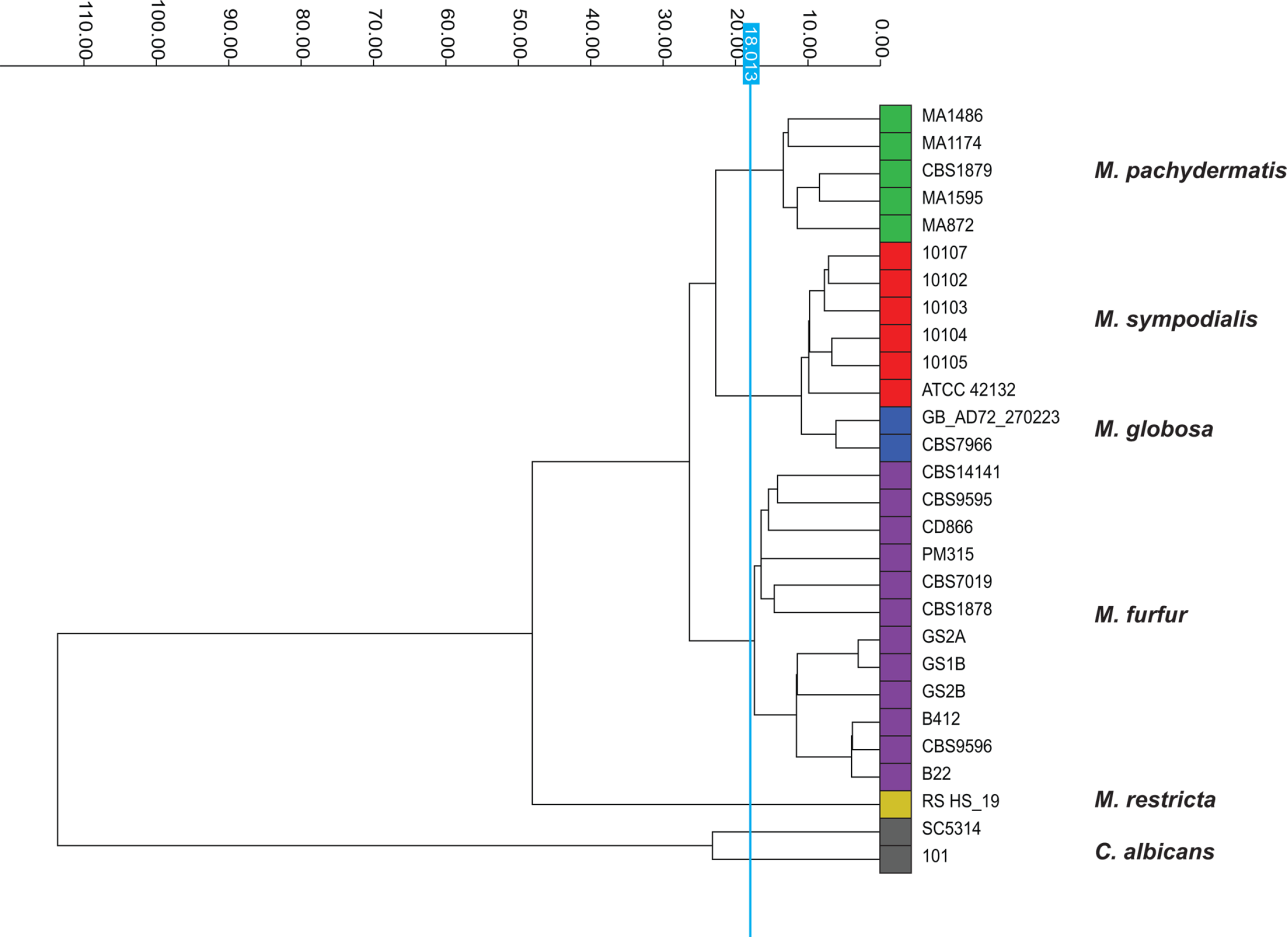
FTIR spectroscopy discriminates between different *Malassezia* spp. Dendrogram generated from clustering FTIR spectra of *Malassezia* spp. and *Candida albicans* to assess species differentiation. Spectra are color-coded by strain and species, with each row representing the average of nine spectra derived from three independent biological repeats. The vertical blue line denotes the clustering cut-off value (18.013). Dimensionality reduction was performed using linear discriminant analysis (LDA; 27 LDs of 30 PCs; 99.8% variance) followed by hierarchical cluster analysis using Euclidean distance and average linkage, providing an overview of FTIR-based phenotypic relationships among isolates.

After establishing a robust protocol for applying FTIR spectroscopy to *Malassezia*, we investigated the discriminatory power of the method at both the genus and species level. For this, we compared the FTIR spectra of several of the most prevalent and clinically relevant *Malassezia* spp. We also included two *C. albicans* strains in the analysis, which clustered distantly from *Malassezia* spp. (Fig. 3). Within the *Malassezia* genus, individual species clustered distinctly from each other (Fig. 3), confirming the potential of FTIR for species-level differentiation. Within species, different strains were clearly segregated from each other, with biological and technical replicates always clustering most closely (Fig. 3).

### FTIR spectroscopy achieves strain-level resolution in *Malassezia pachydermatis*

**Fig 4:**
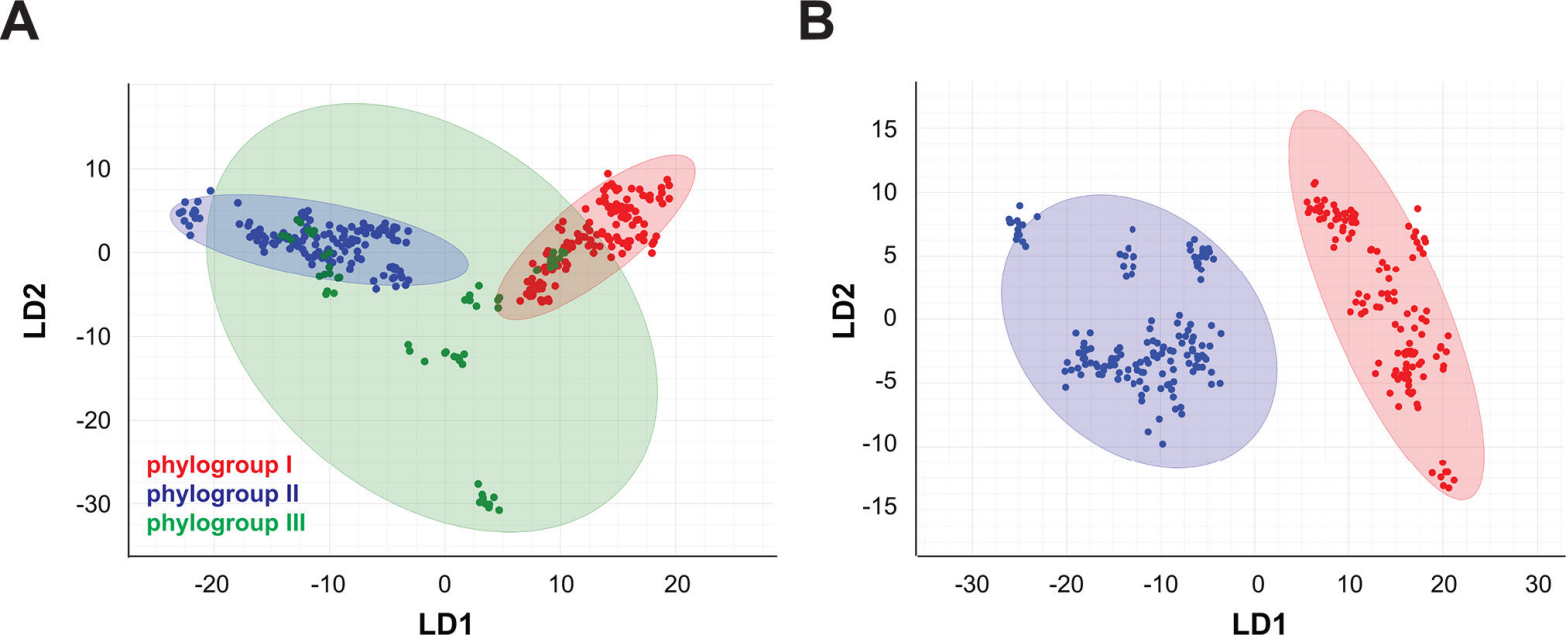
FTIR spectroscopy achieves strain-level resolution in *M. pachydermatis*. Scatter plots showing the distribution of *M. pachydermatis* phylogroups I (*n* = 12), II (*n* = 12), and III (*n* = 6) strains (**A**), and phylogroups I (*n* = 12) and II (*n* = 12) strains (**B**) within the FTIR spectral space. Each strain is represented by nine spectra (•) pooled from three independent biological replicates with three technical replicates each. Data were processed using LDA based on 30 principal components. The diagram displays the first two LD axes, with spectra color-coded by phylogroup (I: red, II: blue, and III: green).

**Fig 5:**
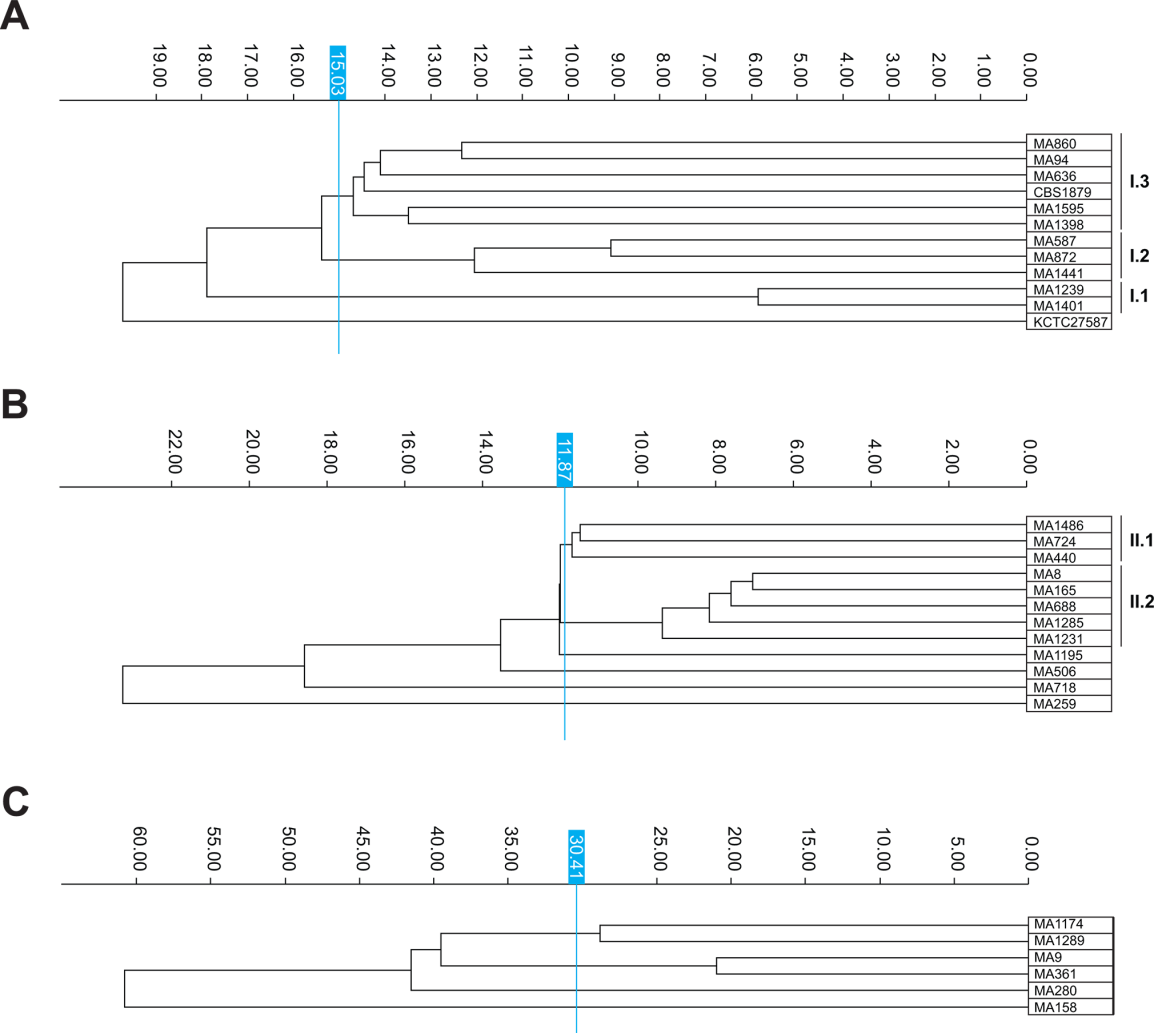
Subclusters in phylogroups I, II, and III based on FTIR spectral profiles. Dendrogram generated from clustering FTIR spectra of *M. pachydermatis* strains of phylogroups I, II, and III separately to assess intra-phylogroup variability. Each row represents the average spectrum of a strain, calculated from at least nine individual absorption spectra (three independent biological replicates with three technical replicates each per strain). The vertical blue lines denote the cut-off value for clustering. Dimensionality reduction: (**A**) phylogroup I: LDA (11 LDs of 30 PCs; 98.7% variance), (**B**) phylogroup II: LDA (11 LDs of 30 PCs; 99.2% variance), and (**C**) phylogroup III: LDA (5 LDs of 30 PCs; 99.7% variance). Subclusters are indicated to the right.

Next, we evaluated the capacity of FTIR spectroscopy to discriminate among *M. pachydermatis* strains, where a larger number of isolates was available, and to assess its effectiveness in differentiating strains belonging to different phylogroups. Analyzing the FTIR spectra of the 30 strains (Table 1) using LDA grouped them into three clusters broadly corresponding to phylogroups I, II, and III (Fig. 4A; Fig. S5). Phylogroups I and II were well separated (Fig. 4B; Fig. S6), while phylogroup III exhibited greater internal variability and partially overlapped with both phylogroups I and II (Fig. 4 and 5). Within phylogroup III, two strains formed a cluster clearly separate from phylogroups I and II, while the remaining four strains (MA9, MA361, MA1289, and MA1174) were positioned closer to either phylogroup I or II strains (Fig. 4A; Fig. S5). These observations indicate that, although FTIR-LDA can generally distinguish among phylogroups, some strains within phylogroup III cannot be unambiguously assigned based solely on this clustering method.

A more detailed analysis of the FTIR spectra, conducted separately for each phylogroup, identified distinct clustering patterns (Fig. 5; Fig. S7). In phylogroup I, three subclusters were identified: subcluster I.1 included strains MA1239 and MA1401; subcluster I.2 comprised strains MA587, MA872, and MA1441; and subcluster I.3 contained strains MA94, MA636, MA860, MA1398, MA1595, and CBS1879. Strain KCTC27587 was positioned more distantly from the other strains of this phylogroup. Pairwise relatedness analysis further showed that strains MA1239 and MA1401 were the most closely related pair (spectral distance 5.85), followed by MA587 and MA872 (spectral distance 9.08). In phylogroup II, two subclusters were observed: subcluster II.1 included strains MA440, MA724, and MA1486, while subcluster II.2 comprised strains MA8, MA165, MA688, MA1231, and MA1285. Four strains (MA259, MA506, MA718, and MA1195) did not cluster within either of these subgroups. Phylogroup III exhibited the highest intra-group variability, with no clear subclusters observed (Fig. S7C).

### Comparison of the strain clustering based on FTIR spectroscopy vs. WGS-based clustering

We next compared FTIR clustering to WGS, the gold standard for microbial classification, using three metrics: SID, ARI, and AWC. Overall, WGS exhibited slightly higher diversity (SID: 0.662, 95% CI: 0.604–0.720) compared to FTIR (SID: 0.605, 95% CI: 0.527–0.682). FTIR reproduced the WGS-based clustering of phylogroup I (*n* = 12) and II (*n* = 12) strains with perfect agreement, as reflected by ARI and AWC values of 1.00. Notably, FTIR clearly distinguished phylogroups I and II with high confidence, despite these groups being more similar to each other based on ANI (mean divergence: I vs. II = 3.63%, I vs. III = 3.68%, II vs. III = 4.26%). Furthermore, phylogroup III, which showed the least diversity by WGS, exhibited the highest diversity in FTIR profiles, suggesting that FTIR captures phenotypic variation not evident in genomic comparisons. In the case of phylogroup III strains, FTIR misclassified three strains, resulting in an overall ARI of 0.768 when considering all studied strains (*n* = 30). Despite this misclassification, the AWC indicated that FTIR clusters were highly predictive of WGS clusters (AWC: 0.876, 95% CI: 0.824–0.929), highlighting FTIR’s potential as a reliable alternative method. The reverse predictive power, from WGS to FTIR, was slightly lower (AWC: 0.684, 95% CI: 0.406–0.962), reflecting the greater resolution of WGS. These findings suggest that while FTIR cannot fully replace WGS, it provides a reliable approximation of WGS-based classifications and a valuable first-line screening option or an alternative to WGS in settings where WGS is limited by cost or turnaround time.

### Establishing an FTIR-based classifier for *M. pachydermatis*

Using the IR Biotyper software, we trained an ANN model to classify haploid *M. pachydermatis* strains based on their FTIR spectra. The training set consisted of 139 spectra from 13 strains representing all three phylogroups, with multiple biological and technical replicates per strain to capture within-group variation (Fig. S1). For validation, 153 spectra from 17 additional strains, also spanning all phylogroups, were withheld from training and used to assess model performance. After 142 training cycles, the model achieved 100% accuracy on the training set (Fig. S8).

Post validation, the automated classifier performance was evaluated using sensitivity, specificity, error rate, and predictive values. Validation results showed perfect classification for phylogroups I and II, with 108/108 spectra correctly identified, yielding 100% accuracy and sensitivity for these groups (Tables 3 and 4; Table S6). For phylogroup III, 43 out of 54 spectra were correctly classified (79.6% sensitivity), while the remaining 11 spectra from two strains were misclassified as either phylogroup I (*n* = 5) or phylogroup II (*n* = 6). These misclassifications lowered specificity for phylogroup I to 96.9% and phylogroup II to 96.3%, while specificity for phylogroup III remained at 100% (no false positives). Positive predictive values were high across groups—95.6% for phylogroup I, 94.7% for phylogroup II, and 100% for phylogroup III—as were negative predictive values (100% for phylogroups I and II, and 95.2% for phylogroup III).

**TABLE 3:** Confusion matrix showing classification performance of the IR Biotyper ANN model across *M. pachydermatis* phylogroups

Predicted/actual	Phylogroup I	Phylogroup II	Phylogroup III
Phylogroup I	**108^*b*^**	0	5
Phylogroup II	0	**108**	6
Phylogroup III	0	0	**43**
Misclassified^*a*^	0	0	11

^*a*^ Misclassified spectra in phylogroup III, suggesting some spectral overlap with phylogroups I and II among some strains.

^*b*^ Bold values denote spectra correctly classified.

**TABLE 4:** Classifier performance metrics per phylogroup

Parameter^*a*^	Phylogroup I	Phylogroup II	Phylogroup III
Accuracy (%)	100 (108/108)	100 (108/108)	79.6 (43/54)
Error rate (%)	0 (0/108)	0 (0/108)	20.4 (11/54)
Sensitivity (%)	100 (108/108)	100 (108/108)	79.6 (43/54)
Specificity (%)	96.9 (157/162)	96.3 (156/162)	100 (216/216)
Positive predictive value (%)	95.6 (108/113)	94.7 (108/114)	100 (43/43)
Negative predictive value (%)	100 (157/157)	100 (156/156)	95.2 (216/227)

^*a*^ Data derived from phylogroup I (*n* = 12), II (*n *= 12), and III (*n *= 6) strains, with nine spectra per strain. The reported accuracy and error rate are for each individual phylogroup.

**Fig 6:**
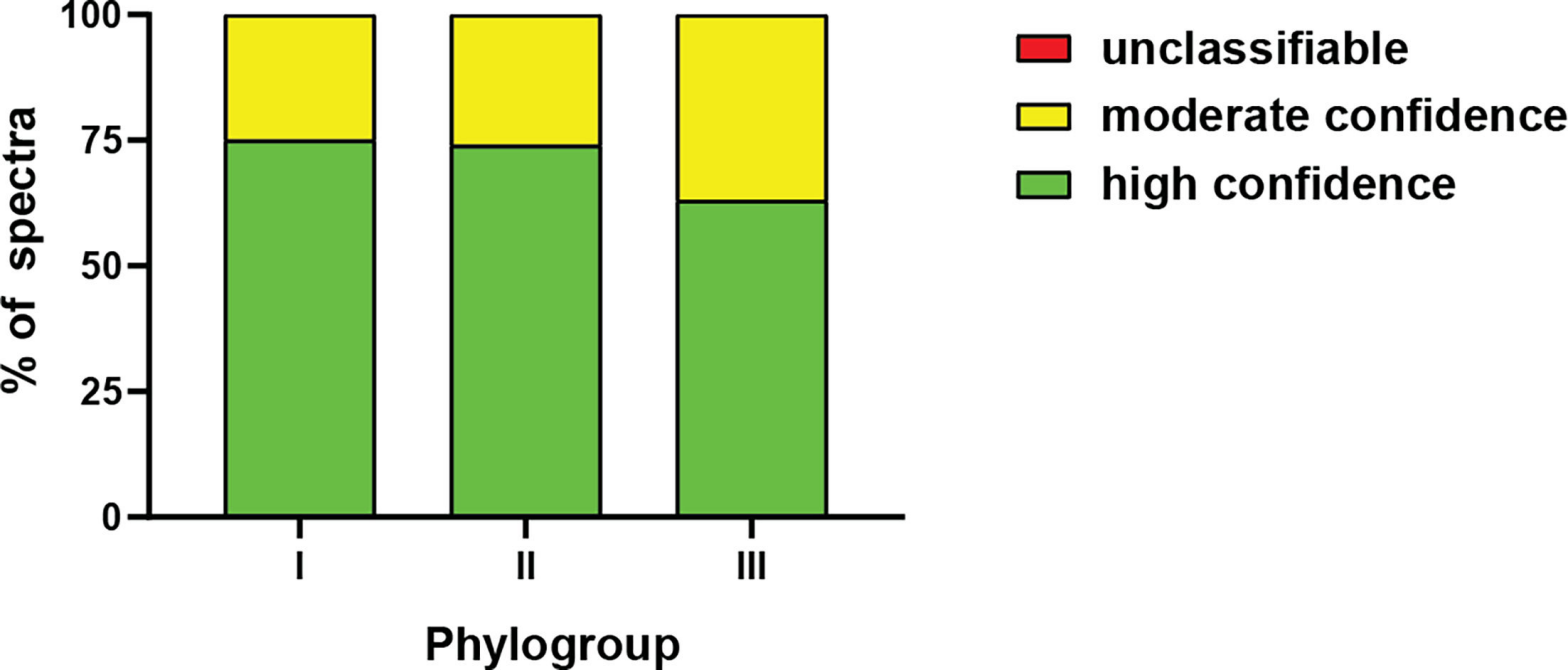
Classification confidence per phylogroup. The bar graph shows the percentage of spectra for each phylogroup, classified as green, yellow, or red, relative to the total number of spectra per phylogroup, from a minimum of nine individual absorption spectra (three independent biological replicates with three technical replicates each per strain). Classification scores are color-coded; green (<1.0) indicates spectra within the classifier’s training set boundaries and considered reliable, yellow (1.0–2.0) represents spectra near the boundaries and considered moderately reliable, and red (>2.0) indicates spectra outside the training set boundaries, deemed unreliable.

Classifier confidence was further evaluated using the IR Biotyper traffic light color-coding system, which categorizes outlier values as green (<1.0, high confidence), yellow (1.0–2.0, moderate confidence), and red (>2.0, unclassifiable spectra). Of the 270 tested spectra, 188 (69.6%) received green scores, and 82 (30.4%) received yellow scores (moderate confidence), with no spectra falling into the red category. Performance was similar for phylogroups I and II, while phylogroup III produced a higher proportion of yellow scores (Fig. 6). Except for two misclassified strains from phylogroup III, the classifier consistently assigned green or yellow scores to all biological replicates of each strain. These misclassifications highlight the need for further refinement of the classifier for this subgroup; however, additional phylogroup III isolates were not available to us at the time of this study, preventing further optimization. Importantly, the classifier excluded all non-*M*. *pachydermatis* spectra (*n* = 90) using the selected red score threshold, demonstrating its potential not only for intra-species strain typing but also for robust species-level exclusion. Overall, the model achieved sensitivity and accuracy of 95.9% (259/270), error rate 4.1% (11/270), and specificity of 98.0% (529/540) across all phylogroups. Together, these findings indicate that the FTIR-based classifier has strong potential for *M. pachydermatis* strain typing, broader diagnostic applications, and robust exclusion of unrelated species.

## DISCUSSION

This study presents a novel FTIR spectroscopy protocol for analyzing diversity in the skin commensal yeast *Malassezia* using the IR Biotyper. Our findings expand upon and confirm an earlier report that used FTIR spectroscopy to differentiate *M. furfur*, *M. sympodialis*, *M. obtusa*, *M. slooffiae*, and *M. globosa* (52). Here, we validate FTIR spectroscopy as a reliable, rapid, and cost-effective alternative to conventional culture-based strain-typing workflows (MLST/WGS) for differentiating *Malassezia* species and strains in both epidemiological research and routine clinical or veterinary applications. FTIR showed a high discriminatory power for the prevalent and clinically relevant *Malassezia* species included in this study, with unsupervised clustering providing a proof-of-concept for species-level differentiation based on FTIR-derived phenotypic profiles. However, species-level discrimination based on hierarchical clustering should be interpreted cautiously, given the limited number of strains analyzed per species other than *M. pachydermatis*. Hierarchical clustering of FTIR data primarily provides an overview of phenotypic relationships and is sensitive to outliers and clustering parameters, rather than a definitive species classifier. Beyond species-level separation, while we have not experimentally assessed additional species, our results suggest that the method has the potential to capture intraspecies diversity more broadly. Future studies, including larger and phylogenetically broader strain collections, will be required to fully evaluate the robustness and generalizability of FTIR-based species identification across the *Malassezia* genus.

The insights gained from this work also enhance our understanding of *M. pachydermatis* diversity and population structure. WGS confirmed the existence of three well-supported phylogroups within *M. pachydermatis*, providing a robust genomic framework against which FTIR-based clustering could be evaluated. Of the three phylogroups, group III appeared to be the most heterogeneous when analyzed by FTIR, although in MLST, WGS, and ANI analyses phylogroup III strains form a coherent clade. The misclassification of some phylogroup III strains by FTIR likely reflects phenotypic breadth rather than phylogenetic ambiguity. Incorporating additional phylogroup III representatives into the training set would likely help stabilize decision boundaries and improve classification performance, suggesting that strains in this group exhibit broader variation in FTIR-detectable biochemical traits, such as cell-wall components or metabolic state.

The protocol we established for FTIR spectroscopy enables reproducible and consistent spectra acquisition, consistent with previous studies demonstrating the reproducibility of FTIR-based approaches for fungal strain typing (53–56). However, reproducibility in FTIR-based fungal strain typing is strongly dependent on standardized cultivation and sample preparation procedures, as spectral profiles are sensitive to factors including growth medium, incubation time, temperature, and physiological state (56–59). Previous work in yeasts such as *Saccharomyces cerevisiae* and *Candida* spp. has further shown that careful control of growth and harvesting conditions is particularly important when analyzing closely related strains (53, 55). Moreover, reproducibility was found to improve when cells were harvested during the late exponential or early stationary phase, indicating that physiological synchronization is essential for minimizing spectral variability (54). In agreement with these observations, all strains in the present study were analyzed under standardized cultivation and sample-processing conditions, which contributed to the strong reproducibility observed across biological and technical replicates. One of the challenges we faced during sample preparation was the poor ability to resuspend *Malassezia* cells in ethanol. We overcame this by first resuspending the fungal colony material in water and then adding ethanol, in contrast to the manufacturer’s standard “ethanol-first” protocol. Our observations suggest that *Malassezia* colonies exhibit high hydrophilicity, which may account for their improved resuspension in water. Overall, our protocol supported homogeneous sample preparation, controlled cell density, and reproducible spectral acquisition, resulting in tight clustering of technical and biological replicates and low intra-strain spectral variance. These clustering patterns were furthermore consistent with strain relationships inferred by molecular methods, indicating that the protocol is sufficiently robust under controlled conditions. Nevertheless, inter-laboratory reproducibility will require further evaluation in future multi-center studies.

The highest discriminatory power, at both the species and strain levels, was obtained from the spectral region corresponding to polysaccharides, suggesting that yeast cellular carbohydrates may vary significantly between *Malassezia* species and strains. This aligns with reported compositional differences in the carbohydrate-rich cell wall of *Malassezia* species (60). The carbohydrate-rich fungal cell wall, constituting mannans, β-glucans, and chitin, is the primary interaction interface with the host and, as such, drives the host cell response to the fungus. The fungal cell wall also plays essential roles in fungal virulence and stress tolerance, with variations in its components influencing immune evasion, tissue adhesion, and biofilm formation. Notably, *Malassezia* cell walls contain abundant branched β-(1, 6)-glucan and chitosan, which distinguishes them from the cell walls of other yeasts like *C. albicans* and *S. cerevisiae* (61, 62). However, relatively little is known about inter- and intraspecies differences in *Malassezia* cell wall composition. Correlating specific FTIR spectral profiles with functional traits could therefore provide important insights into pathogenicity and immune stimulatory capacity and variations thereof across species and strains. Studies in *S. cerevisiae* have shown that FTIR spectral variability induced by growth media is not uniform across spectral regions, with the lipid-associated region (3,200–2,800 cm⁻¹) being comparatively stable, whereas the carbohydrate/fingerprint region (approximately 1,300–700 cm⁻¹) is highly sensitive to both growth conditions and physiological state (53, 54). Therefore, the application of the carbohydrate-rich spectral region for microbial discrimination requires careful standardization of cultivation conditions and growth phase.

Growth conditions can also influence FTIR profiles by altering the micro- and macromolecular composition of cells (55–57). For instance, FTIR-based differentiation of *Pseudomonas aeruginosa* was superior when cells were grown on Mueller–Hinton agar compared to 5% sheep blood agar (63, 64), and greater reproducibility and discriminatory power were demonstrated for *Lactiplantibacillus plantarum* when cultured in liquid media rather than on agar (65). Among the five liquid media tested (56), Yeast Malt Extract broth (YMB) provided the highest discriminatory power for most food-associated yeast genera, highlighting the strong influence of growth medium on FTIR-based classification performance. In *Malassezia*, media other than mDixon, the standard medium routinely used for culture and employed in our study, may alter the discriminatory power of FTIR spectroscopy. When considering alternative growth conditions for *Malassezia*, media simulating different skin environments, including healthy versus barrier-impaired and inflamed skin differing in pH, salt concentration, and lipid composition, should be explored (66–69). Because skin conditions are known to influence *Malassezia* growth and gene expression profiles (70, 71), they may also shape FTIR-detectable traits. This raises the possibility of applying FTIR to discriminate isolates from healthy versus diseased skin, which, if causally linked to pathogenicity, could serve as a predictive tool for disease progression or clinical outcome in atopic dermatitis and other *Malassezia*-associated diseases. Our study further suggests that the FTIR approach has great potential if it is extended to other clinically important fungi in the future.

While FTIR does not eliminate the need for culturing and therefore does not outperform direct molecular assays in terms of time-to-result (72, 73), strain-level characterization of *Malassezia* by MLST or WGS is currently also almost exclusively performed on cultured isolates. Culture-independent, strain-level characterization of *Malassezia* directly from samples remains challenging due to the low biomass of skin samples, frequent host DNA contamination, resistance of its complex cell wall to standard lysis methods, and primer mismatches that lead to underrepresentation in amplicon-based approaches. In this context, FTIR offers advantages in scalability, cost per sample, and ease of automation once isolates are obtained, making it particularly attractive for strain typing in epidemiological surveillance and large-scale veterinary or clinical applications. FTIR can serve as a high-throughput first-line typing tool, reserving genomic approaches for selected isolates requiring higher-resolution analysis.

The *M. pachydermatis* strains included in our study are limited to haploid strains. In other *Malassezia* species, hybrid strains with chimeric genomes presumably arising from mating between haploid lineages have been reported (40, 74). Hybridization can alter gene dosage, allele combinations, and regulatory networks and may be accompanied by aneuploidy or segmental gene copy-number variation. Such changes could modify cell surface composition, lipid metabolism, and stress responses, all of which contribute to FTIR spectra. Whether hybrids occur in *M. pachydermatis* and whether their FTIR signatures are distinguishable from those of haploid strains remains to be determined. Addressing this will require confirmed hybrid isolates, matched culture conditions and growth phases, and joint analyses that relate FTIR clustering to whole-genome features such as ploidy, copy-number state, and parental haplotype composition. If hybrids are present and display distinct FTIR profiles, this would refine the classifier and further clarify how genomic context shapes the phenotypes captured by FTIR.

The development of an FTIR-based classifier for *M. pachydermatis* represents a significant advancement in the study of *Malassezia*, particularly given the limitations of traditional phenotypic identification methods. This approach provided valuable insights into within-species molecular diversity and phylogenetic relationships among strains, despite the modest training set. In contrast to genotypic methods, a key limitation of FTIR and other phenotypic approaches is their sensitivity to phenotypic changes resulting from minor genetic mutations, which can influence classification even among closely related or clonally derived strains. Expanding the training data to include a broader range of phylogenetically diverse strains and geographic origins will likely enhance the performance and applicability of the classifier across diverse settings. Looking ahead, FTIR may offer a rapid and accurate method for detecting potentially pathogenic *M. pachydermatis* strains, with implications for human and animal health. Applying our FTIR-ANN pipeline on a larger prospective collection of clinical isolates will be essential to bridge the gap between this methodological proof-of-concept and real-world use (75). Realizing the full potential of the approach will require further optimization and validation in multi-center cohorts, the application of supervised FTIR-based classifiers once sufficiently large and balanced training data sets become available, integration with WGS to identify the genomic determinants underlying FTIR-detected phenotypic differences, and future exploration of whether the FTIR-ANN pipeline can be applied directly to primary clinical specimens without prior culture.

## Data Availability

All other raw data linked to this study are publicly available at https://doi.org/10.5281/zenodo.20769482. Raw sequencing reads and genome assemblies were deposited in the DDBJ/EMBL/GenBank databases under BioProject PRJNA1330337 (detailed in Table S5).

## SUPPLEMENTAL MATERIAL

Supplemental materials jcm.01422-25-s0001.docx Figures S1 to S8, and Tables S4 and S6.

Supplemental tables jcm.01422-25-s0002.xlsx Tables S1 to S3, and S5.

ASM does not own the copyrights to Supplemental Material that may be linked to, or accessed through, an article. The authors have granted ASM a non-exclusive, world-wide license to publish the Supplemental Material files. Please contact the corresponding author directly for reuse.

## References

[1] Puig L, Bragulat MR, Castellá G, Cabañes FJ. 2017. Characterization of the species *Malassezia pachydermatis* and re-evaluation of its lipid dependence using a synthetic agar medium. PLoS One 12:e0179148. doi:10.1371/journal.pone.0179148 28586389 PMC5460872

[2] Guillot J, Bond R. 2020. *Malassezia* yeasts in veterinary dermatology: an updated overview. Front Cell Infect Microbiol 10:79. doi:10.3389/fcimb.2020.00079 32181160 PMC7059102

[3] Ianiri G, LeibundGut-Landmann S, Dawson TL. 2022. *Malassezia*: a commensal, pathogen, and mutualist of human and animal skin. Annu Rev Microbiol 76:757–782. doi:10.1146/annurev-micro-040820-010114 36075093

[4] Gushiken-Ibañez E, Stokmaier M, Barone G, Staropoli A, Karakaya T, Beer D, Vinale F, Ianiri G, LeibundGut-Landmann S. 2025. The skin commensal yeast *Malassezia* promotes tissue homeostasis via the aryl hydrocarbon receptor. bioRxiv. doi:10.1101/2025.03.26.645515 PMC13323057 42321537

[5] Kowalski CH, Nguyen UT, Lawhorn S, Smith TJ, Corrigan RM, Suh WS, Kalan L, Barber MF. 2025. Skin mycobiota-mediated antagonism against *Staphylococcus aureus* through a modified fatty acid. Curr Biol 35:2266–2281. doi:10.1016/j.cub.2025.03.055 40233753 PMC12093282

[6] Li H, Goh BN, Teh WK, Jiang Z, Goh JPZ, Goh A, Wu G, Hoon SS, Raida M, Camattari A, Yang L, O’Donoghue AJ, Dawson TL. 2018. Skin commensal *Malassezia globosa* secreted protease attenuates *Staphylococcus aureus* biofilm formation. J Invest Dermatol 138:1137–1145. doi:10.1016/j.jid.2017.11.034 29246799

[7] Lorch JM, Palmer JM, Vanderwolf KJ, Schmidt KZ, Verant ML, Weller TJ, Blehert DS. 2018. *Malassezia vespertilionis* sp. nov.: a new cold-tolerant species of yeast isolated from bats. Persoonia 41:56–70. doi:10.3767/persoonia.2018.41.04 30728599 PMC6344816

[8] Theelen B, Cafarchia C, Gaitanis G, Bassukas ID, Boekhout T, Dawson TL Jr. 2018. *Malassezia* ecology, pathophysiology, and treatment. Med Mycol Open Access 56:S10–S25. doi:10.1093/mmy/myx134 29538738

[9] Cabañes FJ, Theelen B, Castellá G, Boekhout T. 2007. Two new lipid-dependent *Malassezia* species from domestic animals. FEMS Yeast Res 7:1064–1076. doi:10.1111/j.1567-1364.2007.00217.x 17367513

[10] Puig L, Castellá G, Cabañes FJ. 2016. Cryptic diversity of *Malassezia pachydermatis* from healthy and diseased domestic animals. Mycopathologia 181:681–688. doi:10.1007/s11046-016-0026-3 27283291

[11] Gao Z, Perez-Perez GI, Chen Y, Blaser MJ. 2010. Quantitation of major human cutaneous bacterial and fungal populations. J Clin Microbiol 48:3575–3581. doi:10.1128/JCM.00597-10 20702672 PMC2953113

[12] Hađina S, Bruvo Mađarić B, Kazazić S, Paradžik T, Reljić S, Pinter L, Huber Đ, Vujaklija D. 2023. *Malassezia pachydermatis* from brown bear: a comprehensive analysis reveals novel genotypes and distribution of all detected variants in domestic and wild animals. Front Microbiol 14:1151107. doi:10.3389/fmicb.2023.1151107 37275156 PMC10236562

[13] Cafarchia C, Otranto D. 2004. Association between phospholipase production by *Malassezia pachydermatis* and skin lesions. J Clin Microbiol 42:4868–4869. doi:10.1128/JCM.42.10.4868-4869.2004 15472366 PMC522356

[14] Hadrich I, Khemekhem N, Neji S, Trablesi H, Ilahi A, Sellami H, Makni F, Ayadi A. 2022. Production and quantification of virulence factors in *Malassezia* species. Pol J Microbiol 71:529–538. doi:10.33073/pjm-2022-047 36473111 PMC9944974

[15] Kobayashi T, Kano R, Nagata M, Hasegawa A, Kamata H. 2011. Genotyping of *Malassezia pachydermatis* isolates from canine healthy skin and atopic dermatitis by internal spacer 1 (IGS1) region analysis. Vet Dermatol 22:401–405. doi:10.1111/j.1365-3164.2011.00961.x 21401740

[16] Machado MLS, Cafarchia C, Otranto D, Ferreira RR, Bianchi SP, Latrofa MS, Parisi A, Ferreiro L. 2010. Genetic variability and phospholipase production of *Malassezia pachydermatis* isolated from dogs with diverse grades of skin lesions. Med Mycol 48:889–892. doi:10.3109/13693780903532080 20105099

[17] Grice EA, Dawson TL. 2017. Host-microbe interactions: *Malassezia* and human skin. Curr Opin Microbiol 40:81–87. doi:10.1016/j.mib.2017.10.024 29141240

[18] Chow NA, Chinn R, Pong A, Schultz K, Kim J, Gade L, Jackson BR, Beer KD, Litvintseva AP. 2020. Use of whole-genome sequencing to detect an outbreak of *Malassezia pachydermatis* infection and colonization in a neonatal intensive care unit—California, 2015–2016. Infect Control Hosp Epidemiol 41:851–853. doi:10.1017/ice.2020.73 32370815

[19] Rhimi W, Theelen B, Boekhout T, Otranto D, Cafarchia C. 2020. *Malassezia* spp. yeasts of emerging concern in fungemia. Front Cell Infect Microbiol 10:370. doi:10.3389/fcimb.2020.00370 32850475 PMC7399178

[20] Peano A, Johnson E, Chiavassa E, Tizzani P, Guillot J, Pasquetti M. 2020. Antifungal resistance regarding *Malassezia pachydermatis*: where are we now? J Fungi (Basel) 6:93. doi:10.3390/jof6020093 32630397 PMC7345795

[21] Aizawa T, Kano R, Nakamura Y, Watanabe S, Hasegawa A. 2001. The genetic diversity of clinical isolates of *Malassezia pachydermatis* from dogs and cats. Med Mycol 39:329–334. doi:10.1080/714031036 11556762

[22] Sugita T, Takeo K, Hama K, Virtudazo E, Takashima M, Nishikawa A, Kucsera J, Dorogi J, Komori S, Nakagaki K, Vollekova A, Slavikova E, Farkas V. 2005. DNA sequence diversity of intergenic spacer 1 region in the non-lipid-dependent species *Malassezia pachydermatis* isolated from animals. Med Mycol 43:21–26. doi:10.1080/1369378042000193185 15712605

[23] Schönherr FA, Sparber F, Kirchner FR, Guiducci E, Trautwein-Weidner K, Gladiator A, Sertour N, Hetzel U, Le GTT, Pavelka N, d’Enfert C, Bougnoux M-E, Corti CF, LeibundGut-Landmann S. 2017. The intraspecies diversity of *C. albicans* triggers qualitatively and temporally distinct host responses that determine the balance between commensalism and pathogenicity. Mucosal Immunol 10:1335–1350. doi:10.1038/mi.2017.2 28176789

[24] Han S-H, Chung T-H, Nam E-H, Park S-H, Hwang C-Y. 2013. Molecular analysis of *Malassezia pachydermatis* isolated from canine skin and ear in Korea. Med Mycol 51:396–404. doi:10.3109/13693786.2012.740575 23167706

[25] Castellá G, Coutinho SDA, Cabañes FJ. 2014. Phylogenetic relationships of *Malassezia* species based on multilocus sequence analysis. Med Mycol 52:1–7. doi:10.3109/13693786.2013.815372 23902157

[26] Triana S, González A, Ohm RA, Wösten HAB, de Cock H, Restrepo S, Celis A. 2015. Draft genome sequence of the animal and human pathogen *Malassezia pachydermatis* strain CBS 1879. Genome Announc 3:e01197-15. doi:10.1128/genomeA.01197-15 26472839 PMC4611691

[27] Novais Â, Freitas AR, Rodrigues C, Peixe L. 2019. Fourier Transform Infrared spectroscopy: unlocking fundamentals and prospects for bacterial strain typing. Eur J Clin Microbiol Infect Dis 38:427–448. doi:10.1007/s10096-018-3431-3 30483997

[28] Muchaamba F, Stephan R. 2024. A comprehensive methodology for microbial strain typing using Fourier-Transform Infrared spectroscopy. Methods Protoc 7:48. doi:10.3390/mps7030048 38921827 PMC11207048

[29] Essendoubi M, Toubas D, Bouzaggou M, Pinon J-M, Manfait M, Sockalingum GD. 2005. Rapid identification of *Candida* species by FT-IR microspectroscopy. Biochim Biophys Acta 1724:239–247. doi:10.1016/j.bbagen.2005.04.019 15951116

[30] Lam LMT, Ismail AA, Lévesque S, Dufresne SF, Cheng MP, Vallières É, Luong M-L, Sedman J, Dufresne PJ. 2022. Multicenter evaluation of attenuated total reflectance Fourier Transform Infrared (ATR-FTIR) spectroscopy-based method for rapid identification of clinically relevant yeasts. J Clin Microbiol 60:e0139821. doi:10.1128/JCM.01398-21 34669460 PMC8769734

[31] Lam LMT, Dufresne PJ, Longtin J, Sedman J, Ismail AA. 2019. Reagent-free identification of clinical yeasts by use of attenuated total reflectance Fourier Transform Infrared spectroscopy. J Clin Microbiol 57:e01739-18. doi:10.1128/JCM.01739-18 30787141 PMC6498014

[32] Curtoni A, Pastrone L, Cordovana M, Bondi A, Piccinini G, Genco M, Bottino P, Polizzi C, Cavallo L, Mandras N, Corcione S, Montrucchio G, Brazzi L, Costa C. 2024. Fourier Transform Infrared spectroscopy application for *Candida auris* outbreak typing in a referral intensive care unit: phylogenetic analysis and clustering cut-off definition. Microorganisms 12:1312. doi:10.3390/microorganisms12071312 39065082 PMC11279149

[33] Medeiros AGJ, Nascimento ALF, Rossato L, Santos DA, Peres NT de A, Neto RG de L, Meis JF, Lima KMG, Bastos RW. 2026. Infrared spectroscopy as a promising tool for diagnosing and typing human pathogenic fungi. Mycoses 69:e70151. doi:10.1111/myc.70151 41542956 PMC12809877

[34] Mohammed YF, Salem EZ, Shahin IMI, Abdo HM, Emam HE, Fawzy M, Abdel Salam MF. 2016. Applicability of Fourier Transform Infrared (FTIR) spectroscopy in rapid identification of some *Candida* and dermatophyte species infections in humans. Int J Dermatol 55:1164–1171. doi:10.1111/ijd.4957 27337493

[35] Erukhimovitch V, Tsror (Lahkim) L, Hazanovsky M, Huleihel M. 2010. Direct identification of potato’s fungal phyto-pathogens by Fourier-Transform Infrared (FTIR) microscopy. Spectroscopy 24:609–619. doi:10.1155/2010/507295

[36] Salman A, Lapidot I, Pomerantz A, Tsror L, Shufan E, Moreh R, Mordechai S, Huleihel M. 2012. Identification of fungal phytopathogens using Fourier Transform Infrared-attenuated total reflection spectroscopy and advanced statistical methods. J Biomed Opt 17:17002. doi:10.1117/1.JBO.17.1.017002 22352668

[37] Díaz L, Castellá G, Bragulat MR, Cabañes FJ. 2023. ERG11 gene variability and azole susceptibility in *Malassezia pachydermatis*. Mycopathologia 188:21–34. doi:10.1007/s11046-022-00696-9 36495417 PMC10169892

[38] Kim M, Cho Y-J, Park M, Choi Y, Hwang SY, Jung WH. 2018. Genomic tandem quadruplication is associated with ketoconazole resistance in *Malassezia pachydermatis*. J Microbiol Biotechnol 28:1937–1945. doi:10.4014/jmb.1810.10019 30562885

[39] Guého E, Midgley G, Guillot J. 1996. The genus *Malassezia* with description of four new species. Antonie Van Leeuwenhoek 69:337–355. doi:10.1007/BF00399623 8836432

[40] Theelen B, Mixão V, Ianiri G, Goh JPZ, Dijksterhuis J, Heitman J, Dawson TL, Gabaldón T, Boekhout T. 2022. Multiple hybridization events punctuate the evolutionary trajectory of *Malassezia furfur*. mBio 13:e0385321. doi:10.1128/mbio.03853-21 35404119 PMC9040865

[41] Gaitanis G, Magiatis P, Stathopoulou K, Bassukas ID, Alexopoulos EC, Velegraki A, Skaltsounis A-L. 2008. AhR ligands, malassezin, and indolo[3,2-b]carbazole are selectively produced by *Malassezia furfur* strains isolated from seborrheic dermatitis. J Invest Dermatol 128:1620–1625. doi:10.1038/sj.jid.5701252 18219281

[42] Magiatis P, Pappas P, Gaitanis G, Mexia N, Melliou E, Galanou M, Vlachos C, Stathopoulou K, Skaltsounis AL, Marselos M, Velegraki A, Denison MS, Bassukas ID. 2013. *Malassezia* yeasts produce a collection of exceptionally potent activators of the Ah (dioxin) receptor detected in diseased human skin. J Invest Dermatol 133:2023–2030. doi:10.1038/jid.2013.92 23448877 PMC3714356

[43] Gueho E, Simmons RB, Pruitt WR, Meyer SA, Ahearn DG. 1987. Association of *Malassezia pachydermatis* with systemic infections of humans. J Clin Microbiol 25:1789–1790. doi:10.1128/jcm.25.9.1789-1790.1987 3654952 PMC269333

[44] Applen Clancey S, Ruchti F, LeibundGut-Landmann S, Heitman J, Ianiri G. 2020. A novel mycovirus evokes transcriptional rewiring in the fungus *Malassezia* and stimulates beta interferon production in macrophages. mBio 11:e01534-20. doi:10.1128/mBio.01534-20 32873760 PMC7468202

[45] Gillum AM, Tsay EYH, Kirsch DR. 1984. Isolation of the *Candida albicans* gene for orotidine-5’-phosphate decarboxylase by complementation of *S. cerevisiae ura3* and *E. coli pyrF* mutations. Mol Gen Genet 198:179–182. doi:10.1007/BF00328721 6394964

[46] Coelho MA, David-Palma M, Shea T, Bowers K, McGinley-Smith S, Mohammad AW, Gnirke A, Yurkov AM, Nowrousian M, Sun S, Cuomo CA, Heitman J. 2024. Comparative genomics of the closely related fungal genera *Cryptococcus* and *Kwoniella* reveals karyotype dynamics and suggests evolutionary mechanisms of pathogenesis. PLoS Biol 22:e3002682. doi:10.1371/journal.pbio.3002682 38843310 PMC11185503

[47] Bankevich A, Nurk S, Antipov D, Gurevich AA, Dvorkin M, Kulikov AS, Lesin VM, Nikolenko SI, Pham S, Prjibelski AD, Pyshkin AV, Sirotkin AV, Vyahhi N, Tesler G, Alekseyev MA, Pevzner PA. 2012. SPAdes: a new genome assembly algorithm and its applications to single-cell sequencing. J Comput Biol 19:455–477. doi:10.1089/cmb.2012.0021 22506599 PMC3342519

[48] Yoon S-H, Ha S-M, Lim J, Kwon S, Chun J. 2017. A large-scale evaluation of algorithms to calculate average nucleotide identity. Antonie Van Leeuwenhoek 110:1281–1286. doi:10.1007/s10482-017-0844-4 28204908

[49] Carriço JA, Silva-Costa C, Melo-Cristino J, Pinto FR, de Lencastre H, Almeida JS, Ramirez M. 2006. Illustration of a common framework for relating multiple typing methods by application to macrolide-resistant *Streptococcus pyogenes*. J Clin Microbiol 44:2524–2532. doi:10.1128/JCM.02536-05 16825375 PMC1489512

[50] Pinto FR, Melo-Cristino J, Ramirez M. 2008. A confidence interval for the wallace coefficient of concordance and its application to microbial typing methods. PLoS One 3:e3696. doi:10.1371/journal.pone.0003696 19002246 PMC2577298

[51] Severiano A, Pinto FR, Ramirez M, Carriço JA. 2011. Adjusted Wallace coefficient as a measure of congruence between typing methods. J Clin Microbiol 49:3997–4000. doi:10.1128/JCM.00624-11 21918028 PMC3209087

[52] Kalinowska-Pujdak A, Schmalreck A, Haustein U-F, Nenoff P. 2006. Speziesdifferenzierung von Hefen der Gattung *Malassezia mittels* Fourier-Transform-Infrarot-Spektroskopie. Hautarzt 57:127–136. doi:10.1007/s00105-005-1018-2 16170542

[53] Kohler A, Böcker U, Shapaval V, Forsmark A, Andersson M, Warringer J, Martens H, Omholt SW, Blomberg A. 2015. High-throughput biochemical fingerprinting of *Saccharomyces cerevisiae* by Fourier transform infrared spectroscopy. PLoS One 10:e0118052. doi:10.1371/journal.pone.0118052 25706524 PMC4338198

[54] Corte L, Antonielli L, Roscini L, Fatichenti F, Cardinali G. 2011. Influence of cell parameters in Fourier transform infrared spectroscopy analysis of whole yeast cells. Analyst 136:2339–2349. doi:10.1039/c0an00515k 21494743

[55] Sandt C, Sockalingum GD, Aubert D, Lepan H, Lepouse C, Jaussaud M, Leon A, Pinon JM, Manfait M, Toubas D. 2003. Use of Fourier-transform infrared spectroscopy for typing of *Candida albicans* strains isolated in intensive care units. J Clin Microbiol 41:954–959. doi:10.1128/JCM.41.3.954-959.2003 12624015 PMC150280

[56] Shapaval V, Walczak B, Gognies S, Møretrø T, Suso HP, Wold Åsli A, Belarbi A, Kohler A. 2013. FTIR spectroscopic characterization of differently cultivated food related yeasts. Analyst 138:4129–4138. doi:10.1039/c3an00304c 23741734

[57] Correa-García S, Bermúdez-Moretti M, Travo A, Déléris G, Forfar I. 2014. FTIR spectroscopic metabolome analysis of lyophilized and fresh *Saccharomyces cerevisiae* yeast cells. Anal Methods 6:1855. doi:10.1039/c3ay42322k

[58] Taha M, Hassan M, Essa S, Tartor Y. 2013. Use of Fourier transform infrared spectroscopy (FTIR) spectroscopy for rapid and accurate identification of yeasts isolated from human and animals. Int J Vet Sci Med 1:15–20. doi:10.1016/j.ijvsm.2013.03.001

[59] Saharan RK, Sharma SC. 2011. FTIR spectroscopy and biochemical investigation of ethanol stressed yeast *Pachysolen tannophilus*. Vib Spectrosc 55:85–89. doi:10.1016/j.vibspec.2010.08.003

[60] Thompson E, Colvin JR. 1970. Composition of the cell wall of *Pityrosporum ovale* (Bizzozero) Castellani and Chalmers. Can J Microbiol 16:263–265. doi:10.1139/m70-048 5462975

[61] Shibata N, Saitoh T, Tadokoro Y, Okawa Y. 2009. The cell wall galactomannan antigen from *Malassezia furfur* and *Malassezia pachydermatis* contains beta-1,6-linked linear galactofuranosyl residues and its detection has diagnostic potential. Microbiology (Reading) 155:3420–3429. doi:10.1099/mic.0.029967-0 19389777

[62] Stalhberger T, Simenel C, Clavaud C, Eijsink VGH, Jourdain R, Delepierre M, Latgé J-P, Breton L, Fontaine T. 2014. Chemical organization of the cell wall polysaccharide core of *Malassezia restricta*. J Biol Chem 289:12647–12656. doi:10.1074/jbc.M113.547034 24627479 PMC4007454

[63] Hu Y, Zhu K, Jin D, Shen W, Liu C, Zhou H, Zhang R. 2023. Evaluation of IR Biotyper for carbapenem-resistant *Pseudomonas aeruginosa* typing and its application potential for the investigation of nosocomial infection. Front Microbiol 14:1068872. doi:10.3389/fmicb.2023.1068872 36846786 PMC9947493

[64] Zendri F, Schmidt V, Mauder N, Loeffler A, Jepson RE, Isgren C, Pinchbeck G, Haldenby S, Timofte D. 2024. Rapid typing of *Klebsiella pneumoniae* and *Pseudomonas aeruginosa* by Fourier-transform Infrared spectroscopy informs infection control in veterinary settings. Front Microbiol 15:1334268. doi:10.3389/fmicb.2024.1334268 38371930 PMC10869444

[65] Deidda F, Cordovana M, Bozzi Cionci N, Graziano T, Di Gioia D, Pane M. 2022. In-process real-time probiotic phenotypic strain identity tracking: the use of Fourier transform infrared spectroscopy. Front Microbiol 13:1052420. doi:10.3389/fmicb.2022.1052420 36569057 PMC9772554

[66] Eberlein-König B, Schäfer T, Huss-Marp J, Darsow U, Möhrenschlager M, Herbert O, Abeck D, Krämer U, Behrendt H, Ring J. 2000. Skin surface pH, stratum corneum hydration, trans-epidermal water loss and skin roughness related to atopic eczema and skin dryness in a population of primary school children. Acta Derm Venereol 80:188–191. doi:10.1080/000155500750042943 10954209

[67] Matthias J, Maul J, Noster R, Meinl H, Chao Y-Y, Gerstenberg H, Jeschke F, Gasparoni G, Welle A, Walter J, Nordström K, Eberhardt K, Renisch D, Donakonda S, Knolle P, Soll D, Grabbe S, Garzorz-Stark N, Eyerich K, Biedermann T, Baumjohann D, Zielinski CE. 2019. Sodium chloride is an ionic checkpoint for human T_H_2 cells and shapes the atopic skin microenvironment. Sci Transl Med 11:eaau0683. doi:10.1126/scitranslmed.aau0683 30787167

[68] Chu H, Kim SM, Zhang K, Wu Z, Lee H, Kim JH, Kim HL, Kim YR, Kim SH, Kim WJ, Lee YW, Lee KH, Liu K-H, Park CO. 2023. Head and neck dermatitis is exacerbated by *Malassezia furfur* colonization, skin barrier disruption, and immune dysregulation. Front Immunol 14:1114321. doi:10.3389/fimmu.2023.1114321 36911720 PMC9992991

[69] Pavel P, Leman G, Hermann M, Ploner C, Eichmann TO, Minzaghi D, Radner FPW, Del Frari B, Gruber R, Dubrac S. 2021. Peroxisomal fatty acid oxidation and glycolysis are triggered in mouse models of lesional atopic dermatitis. JID Innov 1:100033. doi:10.1016/j.xjidi.2021.100033 34909730 PMC8659757

[70] Pianalto KM, Telzrow CL, Brown Harding H, Brooks JT, Granek JA, Gushiken-Ibañez E, LeibundGut-Landmann S, Heitman J, Ianiri G, Alspaugh JA. 2024. *Malassezia* responds to environmental pH signals through the conserved Rim/Pal pathway. mBio 15:e0206024. doi:10.1128/mbio.02060-24 39189745 PMC11481519

[71] Goh JPZ, Ruchti F, Poh SE, Koh WLC, Tan KY, Lim YT, Thng STG, Sobota RM, Hoon SS, Liu C, O’Donoghue AJ, LeibundGut-Landmann S, Oon HH, Li H, Dawson TL Jr. 2022. The human pathobiont *Malassezia furfur* secreted protease Mfsap1 regulates cell dispersal and exacerbates skin inflammation. Proc Natl Acad Sci USA 119:e2212533119. doi:10.1073/pnas.2212533119 36442106 PMC9894114

[72] Eghtedarnejad E, Khajeh S, Zomorodian K, Ghasemi Z, Yazdanpanah S, Motamedi M. 2023. Direct molecular analysis of *Malassezia* species from the clinical samples of patients with pityriasis versicolor. Curr Med Mycol 9:28–31. doi:10.18502/CMM.2023.345029.1398 37867590 PMC10590189

[73] Gholami M, Mokhtari F, Mohammadi R. 2020. Identification of *Malassezia* species using direct PCR- sequencing on clinical samples from patients with pityriasis versicolor and seborrheic dermatitis. Curr Med Mycol 6:21–26. doi:10.18502/cmm.6.3.3984 33834139 PMC8018819

[74] Coelho MA, Ianiri G, David-Palma M, Theelen B, Goyal R, Narayanan A, Lorch JM, Sanyal K, Boekhout T, Heitman J. 2023. Frequent transitions in mating-type locus chromosomal organization in *Malassezia* and early steps in sexual reproduction. Proc Natl Acad Sci USA 120. doi:10.1073/pnas.2305094120 PMC10410736 37523560

[75] Kurmann S, Coelho MA, Mertens S, Rostaher A, Fischer N, Martini F, Knecht M, David-Palma M, Heitman J, LeibundGut-Landmann S, Favrot C, Muchaamba F. 2026. Fourier transform infrared spectroscopy reveals high intraspecies diversity of *Malassezia pachydermatis* in dogs with atopic dermatitis. bioRxiv:2026.04.05.716536. doi:10.64898/2026.04.05.716536

