# Supplementary material for: Fourier transform infrared spectroscopy enables rapid strain typing in M. pachydermatis
Source: J Clin Microbiol. 2026 Jul 20;64(8):e01422-25. doi: 10.1128/jcm.01422-25 (PMC13463905; doi:10.1128/jcm.01422-25)
Supplement: Supplementary file 1 [file jcm.01422-25-s0001.docx]

**Supplementary Tables**

**Supplementary Table S1.** MLST reference genomes.

**Supplementary Table S2**. Full matrix of ANI values computed for all pairwise genome comparisons among the analyzed *M. pachydermatis* strains.

**Supplementary Table S3**. Within and between phylogroup ANI and divergence metrics.

**Supplementary Table S5**. Genome assembly and information on sequencing data generated in this study.

🡪 see separate Excel file

**Supplementary Table S4**. Classification metrics formulae

| **Metrics** | **Formulae*** |
| --- | --- |
| Accuracy (%): | = ((TP + TN) / (TP + TN + FP + FN)) × 100 |
| Error Rate (%): | = ((FP + FN) / (TP + TN + FP + FN)) × 100 |
| Sensitivity (Recall) (%): | = (TP / (TP + FN)) × 100 |
| Specificity (%): | = (TN / (TN + FP)) × 100 |
| Positive Predictive Value (%): | = (TP / (TP + FP)) × 100 |
| Negative Predictive Value (%): | = (TN / (TN + FN)) × 100 |

* Where: TP = True Positives, TN = True Negatives, FP = False Positives, and FN = False Negatives.

**Supplementary Table S6.** Classifier performance metrics across Phylogroups

| **Parameter*** | **Phylogroup I** | **Phylogroup II** | **Phylogroup III** |
| --- | --- | --- | --- |
| True Positives | 108 | 108 | 43 |
| False Negatives | 0 | 0 | 11 |
| False Positives | 5 (from III) | 6 (from III) | 0 |
| True Negatives | 270 – (108+5) = 157 | 270 – (108 + 6) = 156 | 270 – (43 + 11) = 216 |
| Accuracy (%) | 95.6 (108/113) | 94.7 (108/114) | 79.6 (43/54) |
| Error rate (%) | 4.4 (5/113) | 5.3 (6/114) | 20.4 (11/54) |
| Sensitivity (%) | 100 (108/108) | 100 (108/108) | 79.6 (43/54) |
| Specificity (%) | 96.9 (157/162) | 96.3 (156/162) | 100 (216/216) |
| Positive Predictive Value (%) | 95.6 (108/113) | 94.7 (108/114) | 100 (43/43) |
| Negative Predictive Value (%) | 100 (157/157) | 100 (156/156) | 95.2 (216/227) |

* Data derived from phylogroup I (n=12), II (n=12), and III (n=6) strains, with 9 spectra per strain making a total of 270 spectra.

**Supplementary Figures**

**Supplementary Figure S1**. **Selection of representative training set strains for FTIR-ANN classifier development.** Representative strains were selected based on spectral variability within each phylogroup. Deviation/parallel plots show vector-normalized average spectra derived from at least nine spectra per strain, pooled from three independent biological replicates with three technical replicates each. These plots were used to quantify within-group spectral variation and ensure the training set captured the full range of spectral diversity, including outliers, to support robust model generalization. This selection was performed independently for each phylogroup: (**A**) I, (**B**) II, (**C**) III, and (**D**) the overall average across all phylogroups, to account for phylogroup-specific spectral characteristics.

**Supplementary Figure S2**. **Single-locus MLST phylogenies for allele assignment across *M. pachydermatis* strains.** Maximum likelihood (ML) trees were inferred for each of the four MLST loci with IQ-TREE v2.3.6, applying the best-fit substitution model for each gene as determined by the Bayesian Information Criterion (BIC): (**A**) the D1/D2 domain of the LSU rRNA gene (K2P), (**B**) the ITS region (model: K2P+I), (**C**) *TUB2* (model: HKY+F+I), and (**D**) *CHS2* (model: K2P+I). Each tree includes the 30 *M. pachydermatis* strains of this study along with reference sequences representing previously defined MLST allele types (boldface) obtained from previous studies (**^a^**Puig et al. (1), **^b^**Díaz et al. (2), **^c^**Hađina et al. (3), and **^d^**Puig et al. (4). Alignments were trimmed to match the amplicon regions defined in the original MLST scheme (1), and trees were reconstructed with 10,000 ultrafast bootstrap replicates and SH-aLRT tests for branch support. Phylogenetic trees were midpoint rooted. Scale bars indicate substitutions per site. Roman numerals on the left correspond to MLST allele types previously defined (1–4), while newly inferred types are shown in purple. Colored vertical bars to the right of each tree indicate whole-genome phylogroup classifications (see Figure 1): phylogroup I (red), phylogroup II (blue), and phylogroup III (green), with dashed lines indicating uncertain phylogroup placement for specific reference sequences/strains.

**Supplementary Figure S3**. **Establishment of a working protocol for analyzing *Malassezia* by FTIR.** Comparison of the manufacturer’s recommended ethanol-then-water sample preparation protocol (**A**) versus our modified water-then-ethanol approach (**B**). Shown are the fungal suspension after vortexing (left) and the corresponding sample spots in triplicate on a silicon-based 96-sample plate (right).

**Supplementary Figure S4**. **Identification of the most appropriate splicing method.** Scatter plots showing the distribution of 30 *M. pachydermatis* strains within the FTIR spectral space. Each strain is represented by nine spectra (•), pooled from three independent biological replicates with three technical replicates each. Data were processed using Linear Discriminant Analysis (LDA) based on 30 principal components (PCs) and the spectral area of polysaccharides (1300–800 cm⁻¹, (**A**)). proteins (1800–1500 cm⁻¹, (**B**)), lipids (3000-2800 cm⁻¹, 1500-1400 cm⁻¹, (**C**)), or of combined proteins and polysaccharides (1800–900 cm⁻¹, (**D**)). The diagrams display the first two LD axes, with spectra color-coded by phylogroup.

**Supplementary Figure S5. FTIR spectroscopy discriminates *M. pachydermatis* phylogroup I, II, and III strains.** (**A**) *M. pachydermatis* intra-species diversity. 3D scatter plot showing the distribution of *M. pachydermatis* phylogroup I (n=12), II (n=12), and III (n=6) strains within the FTIR spectral space. Each strain is represented by nine spectra (individual symbols), pooled from three independent biological replicates with three technical replicates each. The data were processed using LDA based on 30 PCs. The diagram displays the first three LD axes, with spectra color-coded by phylogroup (I: red, II: blue, III: green). Wave number region 1300–800 cm⁻¹ (polysaccharides). (**B-C**) Dendrogram generated by clustering FTIR spectra of *M. pachydermatis* strains from phylogroups I, II, and III (**B**) or I and II (**C**). Each row represents the average spectrum of a strain, calculated from a minimum of nine individual absorption spectra (three independent biological replicates with three technical replicates each per strain). Dimensionality reduction was performed using LDA (B: 29 LDs of 30 PCs, capturing 98.9% of variance; C: 23 LDs of 30 PCs, capturing 98.9% of variance). The vertical blue line indicates the clustering cut-off (B: 13.4, C: 17.5) used to define cluster separation. Clustering was performed using hierarchical cluster analysis (HCA) with Euclidean distance and average linkage (UPGMA).

**Supplementary Figure S6.** **FTIR spectroscopy discriminates *M. pachydermatis* phylogroup I and II strains.** (**A**) *M. pachydermatis* intra-species diversity. 3D scatter plots showing the distribution of *M. pachydermatis* phylogroup I (n=12) and II (n=12) strains within the FTIR spectral space. Each strain is represented by nine spectra (individual symbols), pooled from three independent biological replicates with three technical replicates each. Data were processed using LDA based on 30 PCs. The diagram displays the first three LD axes, with spectra color-coded by phylogroup (I: red, II: blue). (**B**) Distance matrix generated by clustering the average FTIR spectra of *M. pachydermatis* phylogroup I and II strains. Dimensionality reduction: LDA based on 23 Linear Discriminants (LDs) of 30 PCs (98.9% variance captured, cut-off value: 17.502, was applied to define cluster separation). Wave number region 1300–800 cm^-1^ (polysaccharides). Clusters are color-coded by phylogroup (I: light gray, II: dark gray), and each row or column in the matrix represents the the pairwise relatedness between two average spectra. Each average spectrum was calculated from at least nine single absorption spectrum per strain, derived from three independent biological replicates with three technical replicates each. Color intensity indicates pairwise spectral relatedness between strains: darker red corresponds to higher relatedness (lower pairwise spectral distance), while darker blue indicates lower relatedness (higher pairwise spectral distance).

**Supplementary Figure S7**. **Subclusters in phylogroups I, II, and III based** **on FTIR spectral profiles**. Distance matrix obtained by clustering the average FTIR spectra of *M. pachydermatis* phylogroup I strains ((**A**), dimensionality reduction: LDA (11 LDs of 30 PCs; 98.7% variance, cut-off value: 15.033), phylogroup II strains ((**B**), dimensionality reduction: LDA (11 LDs of 30 PCs; 99.2% variance, cut-off value: 20.916), and phylogroup III strains ((**C**), dimensionality reduction: LDA (5 LDs of 30 PCs; 99.7% variance, cut-off value: 51.151). Clusters are color-coded. Each row and column in the matrix represent the pairwise relatedness between two average spectra, calculated from at least nine individual absorption spectra per strain (derived from three independent biological replicates with three technical replicates each). Colour intensity indicates pairwise spectral relatedness between strains: darker red corresponds to higher relatedness (lower pairwise spectral distance), while darker blue indicates lower relatedness (higher pairwise spectral distance).

|  | Phylogroup I | Phylogroup II | Phylogroup III | Class recall |
| --- | --- | --- | --- | --- |
| Phylogroup I | 52 |  |  | 100% |
| Phylogroup II |  | 49 |  | 100% |
| Phylogroup III |  |  | 38 | 100% |
| Class precision | 100% | 100% | 100% |  |

**Supplementary Figure S8**. **Confusion matrix showing the output of the trained *M. pachydermatis* classifier.** The diagonal, shaded in green, represents true-positive classifications, with values indicating the number of spectra correctly identified. No misclassifications were observed; all predictions matched the true strain labels after 142 training cycle.

**References**

1.	Puig L, Castellá G, Cabañes FJ. 2016. Cryptic Diversity of Malassezia Pachydermatis from Healthy and Diseased Domestic Animals. Mycopathologia 181:681–688. doi:10.1007/s11046-016-0026-3.

2.	Díaz L, Castellá G, Bragulat MR, Cabañes FJ. 2023. ERG11 Gene Variability and Azole Susceptibility in Malassezia pachydermatis. Mycopathologia 188:21–34. doi:10.1007/s11046-022-00696-9.

3.	Hađina S, Bruvo Mađarić B, Kazazić S, Paradžik T, Reljić S, Pinter L, Huber Đ, Vujaklija D. 2023. Malassezia pachydermatis from brown bear: A comprehensive analysis reveals novel genotypes and distribution of all detected variants in domestic and wild animals. Front Microbiol 14:1151107. doi:10.3389/fmicb.2023.1151107.

4.	Puig L, Bragulat MR, Castellá G, Cabañes FJ. 2017. Characterization of the species Malassezia pachydermatis and re-evaluation of its lipid dependence using a synthetic agar medium. PLoS One 12:e0179148. doi:10.1371/journal.pone.0179148.
